# Prevention of recurrent respiratory infections: Inter-society Consensus

**DOI:** 10.1186/s13052-021-01150-0

**Published:** 2021-10-25

**Authors:** Elena Chiappini, Francesca Santamaria, Gian Luigi Marseglia, Paola Marchisio, Luisa Galli, Renato Cutrera, Maurizio de Martino, Sara Antonini, Paolo Becherucci, Paolo Biasci, Barbara Bortone, Sergio Bottero, Valeria Caldarelli, Fabio Cardinale, Guido Castelli Gattinara, Martina Ciarcià, Daniele Ciofi, Sofia D’Elios, Giuseppe Di Mauro, Mattia Doria, Luciana Indinnimeo, Andrea Lo Vecchio, Francesco Macrì, Roberto Mattina, Vito Leonardo Miniello, Michele Miraglia del Giudice, Guido Morbin, Marco Antonio Motisi, Andrea Novelli, Anna Teresa Palamara, Maria Laura Panatta, Angela Pasinato, Diego Peroni, Katia Perruccio, Giorgio Piacentini, Massimo Pifferi, Lorenzo Pignataro, Emanuela Sitzia, Chiara Tersigni, Sara Torretta, Irene Trambusti, Giulia Trippella, Diletta Valentini, Sandro Valentini, Attilio Varricchio, Maria Carmen Verga, Claudio Vicini, Marco Zecca, Alberto Villani

**Affiliations:** 1 Meyer University Hospital, Department of Health Science, University of Florence, Florence, Italy; 2 Departement of Translational Medical Sciences, Federico II University, Naples, Italy; 3 Department of Pediatrics, University of Pavia IRCCS San Matteo foundation, Pavia, Italy; 4 Fondazione IRCCS Ca’ Granda Ospedale Maggiore Policlinico, Milan, Italy; 5 Respiratory Unit, Academic Pediatric Department, Pediatric Hospital Bambino Gesù IRCCS, Rome, Italy; 6 Family Pediatrician, Local Health Unit, Lastra a Signa, Florence, Italy; 7 Family Pediatrician, Local Health Unit, Livorno, Italy; 8 Airway Surgery Unit, Department of Pediatric Surgery, Bambino Gesù Children’s Hospital, Rome, Italy; 9 Department of Obstetrics Gynaecology and Pediatrics, Azienda USL-IRCCS di Reggio Emilia, Reggio Emilia, Italy; 10 Department of Pediatrics, Pediatric Hospital Giovanni XXIII, University of Bari, Bari, Italy; 11 Institute of child health, Bambino Gesù Children’s Hospital IRCCS, Rome, Italy; 12 Department of Clinical and Experimental Medicine, Section of Paediatrics, University of Pisa, Pisa, Italy; 13 Pediatric Primary Care, National Pediatric Health Care System, Caserta, Italy; 14 Family Pediatrician, Local Health Unit, Chioggia, Venice, Italy; 15 Pediatric Department “Sapienza”, University of Rome, Policlinico Umberto I, Rome, Italy; 16 Department of Pediatrics “Sapienza“, University of Rome, Rome, Italy; 17 Department of Biomedical, Surgical, and Odontoiatric Sciences, Università degli Studi di Milano, Milan, Italy; 18 Department of Paediatrics, Paediatric Hospital Giovanni XXIII, Bari, Italy; 19 Department of Woman and Child and General and Specialized Surgery, University of Campania Luigi Vanvitelli, Naples, Italy; 20 Family Pediatrician, Local Health Unit, Trento, Italy; 21 Department of Public Health and Infectious Diseases, Laboratory Affiliated to Istituto Pasteur Italia-Fondazione Cenci Bolognetti, Sapienza University of Rome, Rome, Italy; 22 Department of Otorhinolaryngology, IRCCS Bambino Gesù Pediatric Hospital, Rome, Italy; 23 Family Pediatrician, Local Health Unit, Torri di Quartesolo, Vicenza, Italy; 24 Pediatric Oncology Hematology, Santa Maria della Misericordia Hospital, Perugia, Italy; 25 Department of Surgical Sciences, Dentistry, Gynecology and Pediatrics, Pediatric Clinic, University of Verona, Verona, Italy; 26 Department of Pediatrics Pulmonology and Allergology Section University Hospital of Pisa, Pisa, Italy; 27 Department of Pediatric and Infectious Disease Unit, Bambino Gesù Children’s Hospital, IRCCS, Rome, Italy; 28 Family Pediatrician, Local Health Unit, Colle Val d’Elsa, Siena, Italy; 29 Department of Otolaryngology, Ospedale San Gennaro, Naples, Italy; 30 Family Pediatrician, Local Health Unit Salerno, Vietri sul Mare, Salerno, Italy; 31 Department of Head-Neck Surgery, Otolaryngology, Head-Neck and Oral Surgery Unit, Morgagni Pierantoni Hospital, Forlì, Italy; 32 Pediatric Hematology-Oncology, IRCCS Policlinico San Matteo, Pavia, Italy

**Keywords:** Recurrent respiratory infections, Children, Immune system, Prevention

## Abstract

Recurrent respiratory infections (RRIs) are a common clinical condition in children, in fact about 25% of children under 1 year and 6% of children during the first 6 years of life have RRIs. In most cases, infections occur with mild clinical manifestations and the frequency of episodes tends to decrease over time with a complete resolution by 12 years of age. However, RRIs significantly reduce child and family quality of life and lead to significant medical and social costs.

Despite the importance of this condition, there is currently no agreed definition of the term RRIs in the literature, especially concerning the frequency and type of infectious episodes to be considered. The aim of this consensus document is to propose an updated definition and provide recommendations with the intent of guiding the physician in the complex process of diagnosis, management and prevention of RRIs.

**Supplementary Information:**

The online version contains supplementary material available at 10.1186/s13052-021-01150-0.

## Introduction

Recurrent respiratory infections (RRIs) are a very common clinical condition in childhood, with an important social and economic impact. It is estimated that about 25% of children under 1 year old and 6% of children during the first 6 years of life have RRIs, making them one of the most common reasons for paediatric medical visits in the early years of life [1–3].

Despite being a benign condition that is likely to gradually improve by the age of 12, it significantly interferes with the child’s well-being and runs up significant medical and social costs. Within the scope of RRIs, the specific definition of recurrence has not yet found consensus in literature; on the contrary, the recurrence of certain specific respiratory diseases is well defined. These include infectious rhinitis [4], which is defined as recurrent when it occurs more than 5 times a year, or acute otitis media, which is classed as recurrent with 3 episodes in 6 months or 4 episodes in 12 months [5].

In the past, a clinical score for the assessment of RRIs was proposed for children, and this was based on the type of infectious episode, its duration, paediatric visits, therapy and absence from the community [1]; cases scoring more than 30 points in 6 months were classified as RRIs. Alternatively, and more recently, a definition that takes into account the different trends in respiratory infections in relation to age has been introduced: to be defined as RRIs, 8 or more infections a year are required in subjects under the age of 3, with 6 or more infections in children over the age of 3 [6].

To guide doctors in the management and prophylaxis of children with RRIs, an intersocietal consensus document including an updated definition of RRIs, a practical diagnostic algorithm and recommendations on the use of possible measures to prevent RRIs in children was developed, based on the analysis of the international scientific literature available, developed using the GRADE method (Grading of Recommendations Assessment, Development and Evaluation). As far as the treatment of individual infectious episodes is concerned, the panel recommends that each individual infection be managed in compliance with the national and international guidelines published for each respiratory disease (e.g., tonsillitis, rhinitis, otitis, etc.).

The full text of the document is available on the SIP website (https://sip.it/2020/10/30/la-prevenzione-delle-infezioni-respiratorie-ricorrenti/) and the websites of the other scientific societies represented.

## Methods

To draw up this Inter-society Consensus a panel of experts in pediatrics, pneumology, allergology, immunology, oncohematology, pediatric infectious diseases, otorhinolaryngology, pharmacology, microbiology, pediatric radiology, territorial public health, nursing sciences, research methodology and epidemiology has been identified by the scientific societies of the disciplines involved: the Italian Society for Paediatrics (SIP), the Italian Federation of Paediatricians (FIMP), the Italian Society of Paediatric Respiratory Diseases (SIMRI), the Italian Society of Pediatric Infectious Disease (SITIP), the Italian Society for Preventive and Social Paediatrics (SIPPS), the Italian Society for Paediatric Allergy and Immunology (SIAIP), the Italian Society of Paediatric Otorhinolaryngology (SIOP), the Italian Association of Paediatric Hematology and Oncology (AIEOP), the Italian Society of Paediatric Primary Care (SICuPP), the Italian Society of Otorhinolaryngology – Head and Neck Surgery (SIO e ChCF), Italian Society of Microbiology (SIM), Italian Society of Chemotherapy (SIC) and the Italian Society of Paediatric Nursing. The research was conducted on Pubmed and Embase and included all kinds of clinical studies in children published, in English and Italian, between 01/01/2009 and 31/12/2019. For each question, the keywords used for the search strategy were identified by members of a subcommittee. The research strategy used was as follows: ((recurrent [All Fields] AND (“respiratory tract infections”[Mesh Terms] OR (“respiratory”[All Fields] AND “tract”[All Fields] AND “infections”[All Fields]) OR “respiratory tract infections”[All Fields] OR (“respiratory”[All Fields] AND “infection”[All Fields]) OR “respiratory infection”[All Fields])) OR (recurrent [All Fields] AND (“respiratory tract infections”[Mesh Terms] OR (“respiratory”[All Fields] AND “tract”[All Fields] AND “infections”[All Fields]) OR “respiratory tract infections”[All Fields] OR (“respiratory”[All Fields] AND “infections”[All Fields]) OR “respiratory infections”[All Fields])) AND (“child”[Mesh Terms] OR “child”[All Fields] OR “children”[All Fields]). Search strings are listed in the Additional file 1. Every study included in the review was assessed in terms of method and content using the GRADE method. The quality assessment of the systematic reviews was carried out using the AMSTAR 2 tool.

## Results

### Panel definition of RRIs

To propose a new definition, the heterogeneity of the studies found in literature was initially assessed; all studies reporting the definition of paediatric RRIs (i.e., observational studies, randomised controlled trials, original studies, reviews and meta-analyses, epidemiological studies), were included. The research strategy allowed the initial identification of 4445 studies. After the evaluation of titles and abstracts by 2 data extractors, independently, 213 full-text were evaluated. Of these, 80 studies met the inclusion criteria and were evaluated for the purposes of the new definition (Additional file 1). All the definitions identified in literature through the research were then considered and the new definition was developed, shared and accepted using the Delphi method (Table 1). The multidisciplinary panel of experts drew up the recommendations reported in Table 2.

**Table 1:** Panel definition of RRIs

The criteria for defining a child with Recurrent Respiratory Infections (RRIs) in paediatric age ^**a,b**^ are:	
▪ **1–3 years**^**c**^**:**	
➢ 6 or more respiratory tract infections (1 of which may be pneumonia, including severe pneumonia) in a year or	
➢ 2 mild cases^d^ of pneumonia confirmed by clinical criteria and/or x-ray in a year	
**▪ 3–6 years**^**c**^**:**	
➢ 5 or more respiratory tract infections (1 of which may be pneumonia, including severe pneumonia) in a year or	
➢ 2 mild cases of pneumonia confirmed by clinical criteria and/or x-ray in a year	
**▪ 6–12 years:**	
➢ 3 or more respiratory tract infections (1 of which may be pneumonia, including severe pneumonia) in a year or	
➢ 2 mild cases of pneumonia confirmed by clinical criteria and/or x-ray in a year	

^**a**^ Children with recurrent infections in one area only (e.g., recurrent rhinosinusitis, recurrent otitis media, recurrent wheezing or recurrent pharyngotonsillitis), with known primary or secondary immunodeficiencies (including IgA deficiency), cystic fibrosis and/or CFTR-pathies, primary ciliary dyskinesia, non-cystic fibrosis-related bronchiectasis, genetic disorders, known cardio-respiratory malformations, neuromuscular disorders and other pre-existing chronic lung diseases were excluded from this definition

^**b**^ This definition does not apply to children under 1 year of age

^**c**^**1–3 years =** from 1 year to 2 years and 11 months**; 3–6 years =** from 3 years to 5 years and 11 months; **6–12 years =** from 6 years to 11 years and 11 months

^**d**^ In accordance with the definition of the *British Thoracic Society,* partially modified

**Table:** 

Mild to moderate pneumonia	Severe pneumonia
Body temperature < 38.5°. Respiratory rate < 50 breaths/min Mild respiratory stress No vomiting	Body temperature > 38.5°. Respiratory rate > 50 breaths/min Severe respiratory distress Lifting of nasal fins Cyanosis Grunting Signs of dehydration Tachycardia Refill time > 2”

**Table 2:** Recommendations

Synthetic Molecules	The evidence available to date does not allow recommendation of the routine use of synthetic molecules for the prevention of RRIs (weak negative recommendation).
	Pidotimod has demonstrated a consistent likelihood of efficacy and can be recommended in selected populations of children, always considering the cost-benefit ratio (weak positive recommendation).
**Probiotics, Prebiotics, Symbiotics, Postbiotics**	In the absence of proof of efficacy, the use of oral probiotic formulations should not be recommended for the prevention of RRIs (**weak negative recommendation**).
	Given the scarcity of supporting evidence, the use of nasal spray formulations containing *Streptococcus salivarius* 24SMB should not be recommended for the prevention of RRIs (**weak negative recommendation**).
	In the absence of proof of efficacy and safety, the use of prebiotics and symbiotics should not be recommended for the prevention of RRIs (**weak negative recommendation**).
	In the absence of proof of efficacy and safety, the use of postbiotics should not be recommended for the prevention of RRIs (**weak negative recommendation**).
**Lysates and bacterial extracts**	The evidence available to date does not allow recommendation of the routine use of bacterial lysates for the prevention of RRIs (**weak negative recommendation**).
	Among the lysates, OM-85 has demonstrated a consistent likelihood of efficacy and can be recommended in selected populations of children, always considering the cost-benefit ratio (**weak positive recommendation**).
**Vitamins and trace elements**	Due to the lack of studies conducted, the heterogeneity of the populations studied, the diversity of dosages, formulations and duration of treatments, zinc and other trace elements should not be used in the prophylaxis of RRIs **(weak negative recommendation).**
	There is no evidence that low levels of vitamin A and vitamin E create a predisposition to respiratory infections in children. There is more evidence that reduced levels of vitamin D are associated with an increased incidence of respiratory infections, particularly viral infections, in the first years of life. The heterogeneity of the populations studied, and the diversity of the outcomes considered mean that it is not possible to recommend the use of vitamin D in the prevention of RRIs. In populations with low socioeconomic status and clearly insufficient levels of vitamin D, and in patients with recurrent acute otitis, there may be a greater likelihood of efficacy in the prevention of RRIs (**weak negative recommendation**). Due to the lack of studies conducted, the heterogeneity and small size of the study populations, and the diversity of dosages and duration of treatment, routine vitamin C supplementation should not be used in the prevention of RRIs (**strong negative recommendation**).
**Complementary/alternative medicines**	The studies currently available on the efficacy of homoeopathy, natural substances and phytotherapy, do not allow recommendations on the use of these products in the prevention of RRIs at this time. This is due, in some cases, to the small number of studies, and, in others, to methodological shortcomings or the fact that they do not include patients of exclusively paediatric age.
**Vaccinations**	There is little evidence regarding the role of influenza and anti-pneumococcal vaccinations specifically for the prevention of RRIs. However, in view of the safety, efficacy and cost-benefit data on the use of these vaccinations, they are still recommended in paediatric age groups (**weak positive recommendation).**
**Nasal therapies with hyaluronic acid, thermal waters and resveratrol**	Based on the limited evidence on nasal therapies with hyaluronic acid, thermal waters and resveratrol for the prevention of RRIs currently available, it is not possible to make a recommendation, but their use is not discouraged.
**Modification of risk factors**	There is little literature on modifying risk factors for the prevention of RRIs, so the evidence currently available does not allow recommendation in this sense. However, limiting exposure to environmental and household pollutants is recommended and exposure to second-hand smoke is strongly discouraged.
**Adeno/Tonsillectomy**	Adeno/Tonsillectomy is not recommended for the reduction of RRIs (**strong negative recommendation**). Adeno/Tonsillectomy is not recommended for the reduction of the number of visits to the doctor for RRIs (**strong negative recommendation**). Adeno/Tonsillectomy is not recommended for the reduction of the number of days of illness (**strong negative recommendation**). As regards the impact of Adeno/Tonsillectomy in reducing the use of respiratory tract medications (including bronchodilators, mucolytics, antihistamines, steroids), no recommendation can be made.
**Antibiotic prophylaxis**	No studies are available on the efficacy of antibiotic prophylaxis in preventing RRIs, so no recommendations can be made. However, in view of the need to promote rational use of antibiotics in order to contain the selection of resistant bacterial strains, reduce costs and reduce adverse events, the panel suggests that antibiotic prophylaxis for the prevention of RRIs should be discouraged.

### Practical management of children with RRIs and clinical algorithm

**Figure 1:**
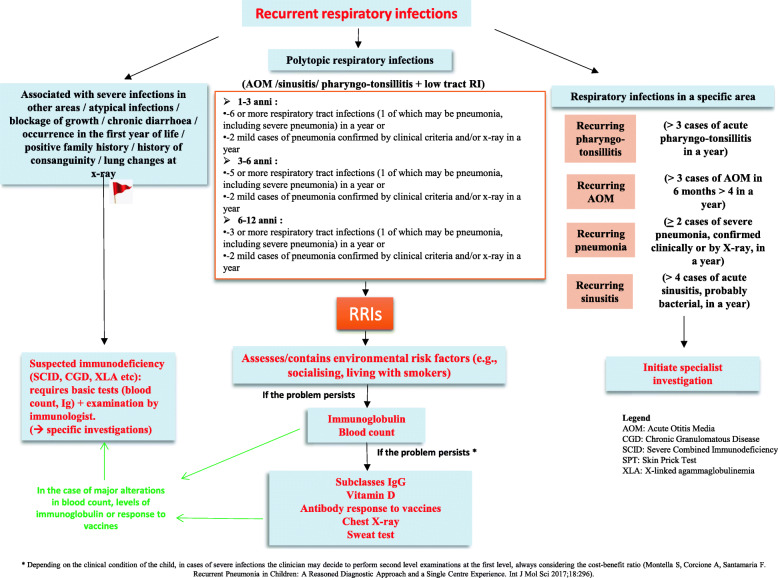
Fig. 1

The diagnosis of RRIs is basically a diagnosis of exclusion of other chronic conditions, such as genetic pathologies, cystic fibrosis, congenital immunodeficiencies, malformities, respiratory pathologies, etc. The personal and family history and a careful objective examination must guide the decision of the paediatrician on the opportunity to subject the child with RRIs to in-depth investigations and the type of examinations to be performed. The panel also drafted the first, second and third level investigations recommended based on the clinical and anamnestic picture and a practical algorithm (Fig. 1). In a child with a positive history of serious and atypical infections, also mediated by opportunistic pathogens, especially with onset in the first months of life, it is important to suspect a primary immunodeficiency disease (PID) and it is necessary to subject the patient to a careful immunological work-up. The Jeffrey Modell Foundation (JMF) defined the warning signs alerting to a suspicion of primary immunodeficiency (PID) in children and adults, based on clinical presentations - mainly infectious diseases - and, in some cases, family history [7, 8]. Equal attention should be paid to a history of reduced growth, chronic diarrhea or other symptoms and signs suggestive for a systemic disease or for a disease related to a specific organ. The diagnostic pathway is different depending on whether it is relapsing infections of the upper and lower respiratory tract. In the case of recurrent infections in a specific area, it will be necessary to undertake a specialist investigation pathway. In the case of recurrent polytopic respiratory infections, once the risk factors have been evaluated and then eliminated, it will be necessary to perform the first and, where appropriate, the second level examinations. In some cases of serious infections such as severe recurrent pneumonia or severe otitis media, the second level examinations can be performed during the first level examinations, always taking into account the risk-benefit ratio [9].

### RRIs prophylaxis (GRADE part)

#### Synthetic molecules

Biological Response Modifiers (BRMs) are substances that can modulate the body’s immunological response through a variety of mechanisms, one of which is the agonist action on Toll-Like Receptors (TLRs), transmembrane receptors that play a key role in the body’s defence, particularly in innate immunity. One mechanism reported for certain BRMs, for example, is to act like TLR agonists, particularly TLR-2 and TLR-4 in bacterial infections, helping enhance the response to microbial infections. Other possible mechanisms include the modulation of interleukin production and stimulation of the adaptive cellular response [10]. Synthetic molecules that can be used in the prevention of RRIs are essentially isoprinosine, levamisole and pidotimod [11]. The studies analysed involve just one synthetic molecule, pidotimod.

Pidotimod is characterised by linear pharmacokinetics that is independent of the type of administration and dose, with a half-life of approximately 4 h. The molecule is rapidly absorbed in the gastrointestinal tract, with a bioavailability of 45%, and is eliminated, unchanged, through the kidney. At a dosage of 400 mg once or twice daily, taken orally on a full stomach, no kinetic differences correlated with patient age were observed [12, 13].

Administration of pidotimod for 90 days in children with Down’s syndrome vaccinated for influenza induced a higher production of specific IgG and a higher ratio of IgG1/lgG3 subclasses than in the control group [10].

A study conducted in children hospitalised for community-acquired pneumonia showed that treatment with pidotimod in combination with antibiotic therapy, compared to antibiotic therapy alone, reduced the potential risk of recurring infection, thanks to *up-regulation* of TLR-2 and increased production of IL-12 and TNF-α with a prolonged effect over time [14].

In a first pilot study, Baraldi et al. assessed the metabolomic profile of 13 children with RRIs, comparing it with that of 15 healthy subjects, and revealed an altered metabolic profile with differences in 138 variables out of 1502 examined. Treatment with pidotimod for 3 months resulted in a rebalancing of 103 altered variables in the metabolomic profile, with the persistence of 35 variables, expressing the functions of the microbiota, which could take longer to return to normal [15]. Subsequently, the same authors conducted a second metabolomic and clinical study in 55 children with RRIs, confirming, in subjects treated with pidotimod, the changes in the metabolomic profile observed in the previous study [16].

Seven studies were selected from the literature review on pidotimod, including 5 low-quality randomised clinical trials (RCTs), assessed using the GRADE method [16–20], 1 systematic review and 1 meta-analysis assessed with AMSTAR II [21, 22], of low and moderate quality respectively.

Licari et al. conducted an unblinded RCT on 100 children between the ages of 3 and 10 with a positive history of RRIs. 45 of these were treated with pidotimod at a dose of 400 mg/day for 60 days and compared with a control group of 44 untreated children. A statistically significant reduction in the number of acute respiratory infections was observed in the children who received pidotimod, compared to the control group, both after 60 days of therapy and in the 2 months of follow-up at the end of treatment. Statistically significant lower drug use was also highlighted in the intervention group at the end of therapy and after the 2 months of follow-up [17].

The same authors also observed a statistically significant difference in the number of medical visits for RRIs, which were found to be lower in children taking pidotimod compared to the control group, with values of 1.3 vs 2.2 per child in the follow-up period (*p* < 0.01) [17]. This is also supported by the review by Esposito et al. [21]. Furthermore, a statistically higher attendance of day-care during the second month of therapy is reported in the group of children treated with pidotimod compared to the control group (79% vs 56%, *p* < 0.01). This statistically significant difference is also confirmed during the 2 months of follow-up, with a school attendance rate of 94% in the intervention group compared to 71% in the control group (*p* < 0.05) [17].

Das et al. enrolled 63 children from an age range of 2–10 years and with a history of RRIs. Of these, 43 were treated with pidotimod (in addition to other acute event therapies) at a dose of 400 mg twice daily for 15 days, and then with pidotimod at a dose of 400 mg once daily for a further 45 days. This intervention group was compared to a control group of 20 children who were given a placebo. At the end of the 6 month follow-up period, several new acute respiratory infectious events of 0.09 + 0.29 were reported in the patients treated with pidotimod vs 2.90 + 0.64 in the group treated with the placebo (*p* = 0.001) [20].

A major difference between the 2 studies, both of which are of low quality, is that the study by Licari et al. excludes children with ongoing acute episodes and pidotimod is administered at a dose of 400 mg a day for 60 days to the intervention group, whereas Das et al. also include children with ongoing respiratory infections and start treatment of the intervention group with pidotimod at a dose of 400 mg twice daily for 15 days and then continue with 400 mg a day for a further 45 days.

In the blind randomised trial by Santamaria et al., 55 children with a history of RRIs were enrolled and divided into 4 groups. The first group was treated with pidotimod at a dose of 400 mg daily and bifidobacteria (B *longum* BB536, 3 × 109 CFU; *B infantis* M-63, 1 × 109 105 CFU; B breve M-16 V, 1 × 109 106 CFU), the second with pidotimod at a dose of 400 mg daily and placebo, the third with placebo and bifidobacteria, and the fourth with placebo only. Treatment was administered 10 days a month for 4 months. During the treatment period and for the following 2 months, the authors did not observe a significant reduction in the number of respiratory tract infections but compared with the placebo-only group, children receiving pidotimod only or in association with bifidobacteria had more symptom-free days (69 vs 44, *p* = 0.003 and 65 vs 44, *p* = 0.02, respectively) and a significant reduction in the percentage of days with symptoms of rhinitis (17% vs 37%, *p* = 0.005 and 15% vs 37%, *p* = 0.004, respectively). The metabolomic analysis showed that children treated with pidotimod (alone or in combination with bifidobacteria) present, respect to children treated with placebo, a biochemical profile characterized by compounds related to the pathway of steroids hormones, hippuric acid and tryptophan. No significant difference in the metabolic profile was found between children receiving bifidobacteria alone and controls [16].

In the moderate-quality meta-analysis by Niu et al., 29 RCTs published up to February 2018 were included, with a total of 4344 children under the age of 14 diagnosed with RRIs. Children treated with pidotimod were compared to control groups treated with placebo or conventional therapy. Taking data from 24 of the 29 RCTs that included the number of RRIs episodes as the study outcome, the authors observed significantly fewer new RRIs episodes in the group treated with pidotimod than in the control group [RR (relative risk) 1.59, CI 95% 1.45–1.74; I^2^ = 51%, *p* < 0.00001]. With regard to the efficacy of pidotimod in reducing the duration of fever and coughing in children with RRIs, the work of Niu et al., taking data from 10 RCTs, showed a statistically significant difference between the group treated with pidotimod and the control group [MD (*mean difference*): − 1.59 days, 95% CI -2.53, − 0.5, I^2^ = 99%, *p* = 0.0009]. This was also confirmed with regard to the duration of fever; analysing 13 RCTs, the authors revealed significantly fewer days with fever in children treated with pidotimod compared to the control group (MD: − 1.68 days, CI 95%-2.12, − 1.24, I^2^ = 99%, *p* < 0.00001) [22]. In the same meta-analysis, taking data from 6 RCTs, significantly reduced use of antibiotics was revealed in the group of children treated with pidotimod compared to the control group, treated with standard therapies only (RR 0.41; 95% CI 0.32–0.1; I^2^ = 40%, *p* < 0.00001) [22].

In the systematic review by Esposito et al., publications from 1997 to 2017 on molecules with an immunomodulating effect on different diseases of the respiratory system in children were analysed. Considering, in particular, studies on the role of pidotimod in RRIs, the authors selected 15 papers investigating the action of the molecule and 9 RCTs assessing clinical outcomes*.* The data suggest that pidotimod can reduce the incidence of infections in children with a history of RRIs, the duration and severity of infectious symptoms, antibiotic use, visits to the doctor and school days lost [21].

Lastly, as regards the efficacy of pidotimod in increasing disease-free days in children with RRIs, the randomised controlled trial by Santamaria et al. reported an increase in the number of disease-free days in the group of children who had received pidotimod associated or otherwise with bifidobacteria compared to children who had received placebo (65 days vs 44 days; *p* = 0.02 and 69 days vs 44 days *p* = 0.03 respectively). No significant difference emerged compared to the group treated with placebo and bifidobacteria [16].

Namazova-Baranov et al., in an unblinded RCT of very low quality, compared 78 children with RRIs treated with pidotimod at a dose of 400 mg/day for 30 days to a control group of 79 children treated with amoxicillin-clavulanic acid; the study reports a significant reduction in the number of RRIs in the group treated with pidotimod compared to the control group [18].

The low-quality study conducted by Walavalkar et al. revealed a statistically significant difference in the number of RRIs in the 2 study groups (amoxicillin-clavulanic acid and pidotimod vs amoxicillin-clavulanic acid and placebo). During the first 15 days of therapy, RRIs occurred in 8.9% of the amoxicillin-clavulanic acid and pidotimod group compared with 66% of the amoxicillin-clavulanic acid and placebo control group (*p* < 0.05). During the next 30 days of maintenance therapy, the authors again reported a statistically significant difference in the number of RRIs, with a value of 1.9% in the group treated with pidotimod compared to 18.2% in the placebo-treated control group (*p* < 0.05). Despite this, there was no statistically significant difference in the number of RRIs in the 2 study groups during the subsequent 6 month follow-up [19].

The datasheet of the product sold in Italy currently indicates the use of this molecule from the age of 3 upwards [23] and in subjects with documented immunodeficiency. In patients with hyper-IgE syndromes, atopic subjects or those with a history of allergic reactions, the preparation should be administered prudently and with caution [24].

The 2 possible and equally recommended dosages, to be taken in both cases away from meals, are:

− 400 mg/day for 2 months in autumn;
− 400 mg × 1–2/day for 10 days a month from October to April.

The safety profile of Pidotimod is good; no serious adverse events were reported in human studies except for one case of suspected Henoch-Schönlein purpura [25]. However, no other association with autoimmune diseases have been reported so far [26].

In conclusion, although the currently available RCTs are limited in number, and most of them of low quality, in limited numbers of children, with outcomes, considered heterogeneous, and using different treatment regimens in terms of duration and dosage, in the majority of cases the data suggest the efficacy of pidotimod in the prevention of RRIs; this could be a monitor for future study design. As a result, the evidence currently available does not allow the recommendation of the use of synthetic molecules routinely for the prevention of RRIs, but in some cases pidotimod can be recommended by the pediatrician, always considering the cost-benefit ratio.

### Probiotics, prebiotics, Symbiotics, Postbiotics

#### The ‘*biomodulators of the gut microbiota*’ [27] are probiotics, prebiotics, symbiotics and postbiotics

The action of certain probiotic strains, live micro-organisms which, taken in adequate quantities, have beneficial effects on the health of the host organism, is expressed by contrasting the growth of pathogenic and pathobiont bacteria, the consolidation of the epithelial barrier function and, above all, immuno-modulating activity [28]. Specific topical probiotic strains (bacteriotherapy) for oral administration (*Streptococcus salivarius* K12, *Lactobacillus salivarius* PS7) or delivery into the pharynx and nasal cavities (*Streptococcus salivarius* 24SMB, *Streptococcus oralis* 89a) have recently been adopted in order to optimise the composition of the relevant microbiota (rhino pharynx, middle ear) and prevent relapsing upper respiratory tract diseases. The different microbial communities that populate the various areas of our body (intestinal, oro-pharyngeal, nasal, pulmonary, cutaneous, urogenital microbiota) are able to “communicate” with the immune system and indirectly with each other through metabolites and cytokines (*crosstalk*) [29].

The recent 2018 Consensus Statement issued by the ISAPP (International Scientific Association for Probiotics and Prebiotics) considers prebiotics to be “substrates used selectively by indigenous microorganisms that can induce beneficial effects on health” [30].

Due to their competitive action on bacterial flora residing in the mucous membranes and to their immunomodulatory action, probiotics have been tested as a tool for prophylaxis and treatment of respiratory infections in various paediatric populations: from the prevention of upper tract infections in infants and children attending day-care centres [31] to the prevention of flare-ups in children with chronic lung diseases such as cystic fibrosis [32].

In 2015, a Cochrane systematic review demonstrated a fair level of efficacy (albeit based on low-quality evidence) of probiotics in reducing the number and mean duration of respiratory infection episodes, as well as antibiotic use, compared to placebo [31]. However, the review by Hao et al. studies the efficacy of these interventions in children, adults and the elderly without distinguishing the efficacy of individual strains and target age. The study by Rautava et al., conducted in children with RRIs, showed no benefit of the probiotic in terms of episode frequency, at least 3 episodes of RRIs, [OR (odds ratio) 0.39, CI 95% 0.11–1.36] or administration of antibiotics [RR (relative risk) 0.71, CI 95% 0.37–1.37] [33].

There are currently no paediatric clinical trials testing the use of probiotics and prebiotics and including a reduction in the frequency or severity of RRIs as the primary outcome.

At the end of the systematic review of literature, only 4 papers met the inclusion/exclusion criteria, 2 of which were RCTs and 2 observational studies assessed using the GRADE method; of these, 3 concerning probiotics and 1 a study for symbiotics. There were no studies on prebiotics and postbiotics (bacterial products or metabolic derivatives of probiotic microorganisms with biological activity for the host).

The low-quality randomised controlled trial by Santamaria et al. tested the efficacy of pidotimod either alone or in association with a mixture of bifidobacteria, in reducing episodes of RRIs in pre-school children (aged 3 to 6). In a subgroup analysis comparing the efficacy of the bifidobacteria mixture taken for the first 10 days of the month for 4 consecutive months, no difference is demonstrated in the number of upper or lower respiratory tract infections or in the number of disease-free days compared to placebo [16].

Nasal administration of a mixture containing *Streptococcus salivarius* 24SMB and *Streptococcus oralis* 89a was correlated with a reduction in the number of RRIs compared to the previous year (2.75 vs 5.98 episodes/year, *p* = 0.0001), the number of school days (2.80 vs 4.50 days/month, p = 0.0001) and workdays lost (1.48 vs 2.33 days/month, p = 0.0001) [34].

In a very low-quality clinical trial, the efficacy of a combination of *Lactobacillus rhamnosus* GG, LC705, *Bifidobacterium breve* 99, *Propionibacterium freudenreichii* JS, taken for 24 months, in reducing the incidence and recurrence of AOM (primary outcome) in children aged 10 months to 6 years was investigated; a reduction in patients with > 4 episodes of RRIs (OR 0.56, CI 95% 0.31–0.99, *p* = 0.046) and > 6 episodes (OR 0.59, CI 95% 0.34–1.03, p = ns) was found [35].

The quality of evidence on the use of symbiotics, a combination of prebiotics and probiotics, in the prevention of RRIs is very low and limited to an observational study (*n* = 167 children) showing the efficacy of Sinerga, a nutritional product containing palmitoylethanolamide, bovine colostrum, phenylethylamine and kluyveromyces FM B0399*,* in reducing the frequency of episodes of airway infection and the prescription of antibiotics, through the down-regulation of the mast cells and the direct and indirect contribution of various immune and growth factors [36].

In conclusion, as regards the role of probiotics in the prevention of RRIs, evidence supporting the use of bifidobacteria or lactobacillus formulations is currently limited to single studies that do not demonstrate significant efficacy, and evidence supporting the use of *Streptococcus salivarius* 24SMB and *Streptococcus oralis* 89a formulations is currently limited to a single low-quality study. Consequently, the evidence currently available does not allow recommendation of the use of probiotics routinely for the prevention of RRIs.

### Lysates and bacterial extracts

Bacterial extracts can be conventionally divided into first-generation extracts containing whole killed bacteria or their lysates, and second-generation extracts containing more immunogenic bacterial components (e.g., ribosomes or proteoglycans) [37].

Regarding their action, it is believed that bacterial extracts can activate both mechanisms of innate immunity and adaptive immunity.

At the end of the selection process, 19 articles were included, including 5 papers (1 meta-analysis and 4 systematic reviews) assessed using the AMSTAR 2 tool, 3 RCTs and 1 retrospective study assessed using the GRADE method, 9 narrative reviews and 1 prospective observational study.

Among the studies included, there is only one concerning the efficacy of Ribomunyl [38]; this study shows positive data and is of moderate quality. The data, however, are insufficient to recommend its use.

Only one study, a low-quality meta-analysis [39], was also included for *polyvalent mechanical bacterial lysate* (PMBL), and although it provides encouraging data, they are insufficient to recommend its use.

With regard to D53, only data published in 2013 in the review by Del-Rio-Navarro et al., referring to earlier studies (published between 1995 and 1885) and seeming promising, are available. However, no post-1995 studies were found, and this product is not currently marketed in Italy, so its use is not recommended.

As regards OM-85, several low to moderate-quality studies were found, all produced by the same research group [40–42], along with 4 systematic reviews of low (*n* = 2), high (*n* = 1), and moderate (*n* = 1) quality [21, 43–45], containing some positive data.

In the very low-quality single-blind RCT conducted in 2014, Esposito et al. considered 68 children aged 3 to 5, with RRIs, vaccinated for influenza, and compared the 33 treated with OM-85, at a dosage of 3.5 mg a day for 10 days a month for 3 months, with the 35 untreated children. The authors reported a statistically significant higher incidence of airway infections in the control group than in the treated group; in particular, the proportions of subjects with at least one episode of upper airway infection were 88.6% vs 60.6%, while those with at least one episode of lower airway infection (acute bronchitis, wheezing and pneumonia) were 42.9% vs 15.2% (*p* < 0.05) respectively. There was also a statistically significant reduction in the mean number of courses of antibiotics administered to treated versus untreated children (0.49 ± 1.06 vs. 1.76 ± 0.63, respectively) and in the mean number of school days lost in treated versus untreated children (3.16 ± 2.10 vs. 6.55 ± 2.34) [40].

In 2019, the same research group conducted a further retrospective study of moderate quality including 400 children aged 3 to 6, with RRIs, of whom 200 were treated with OM-85 at a dose of 3.5 mg per day for 10 days a month for 3 months, for 2 consecutive years, compared with a control group of 200 children with similar clinical characteristics who were not treated. The authors reported a statistically significant higher incidence of airway infections in the control group than in the treated group. New episodes of respiratory infection were diagnosed in about two-thirds of the untreated children, and in only about one-third of the children treated with OM-85, with a reduction of about 50% in the risk of new episodes of RRIs. Similar results were obtained considering the total number of respiratory infections, the number of upper and lower airway infections and the number of cases with wheezing. In the first year of treatment, in particular, the proportion of children with at least one episode of respiratory infection was 36% in the treated children vs 64% in the control group (*p* < 0.05). Similar values were observed in the second year of treatment (33% vs 60%; *p* < 0.05). Eleven children (5.5%) reported mild and transient adverse events to OM-85 during the first year of treatment (5 diarrhoea, 3 vomitings, 2 fever, 1 asthenia) and 9 (4.5%) during the second year (4 diarrhoea, 2 vomiting, 2 headache, 1 asthenia)*.* The study also reports a statistically significantly higher proportion of children treated with antibiotics in the control group than in the group treated with OM-85- (*p* < 0.05), both in the first and second years of the study [41].

In 2019, the same research group published a randomised phase IV, placebo-controlled, double-blind, single-centre, moderate-quality study. In the study, the efficacy of OM-85 was assessed in 288 children aged 1 to 6, with a history of RRIs (123 treated with OM-85 at a dosage of 3.5 mg a day for 10 days a month for 3 months, 41 children treated with OM-85 at 3.5 mg a day for 10 days a month for 6 months, and 124 children, in the control group, who received placebo for 10 days a month for 6 months). On the day of enrolment, 35.8% of the children in the first group, 34.6% in the second group and 36.5% in the third group were vaccinated for influenza with trivalent inactivated vaccine (Fluarix). The number of respiratory tract infections and the number of children with at least one episode of airway infection were significantly lower in the group of children treated with OM-85 for 3 months than in the placebo group (33% vs 65%; *p* < 0.0001). These differences were statistically significant for upper airway infections such as rhinitis, pharyngitis and acute otitis media (*p* < 0.0001 and *p* = 0.006, respectively). A significant reduction in the administration of antibiotics was also observed in the group of children treated with OM-85 compared to the control group (25% vs 50.5%; *p* = 0.0002). The authors also showed a statistically significant reduction (*p* = 0.007) in the mean number of school days lost in treated children compared to those who were untreated (5.10 ± 1.33 vs 4.49 ± 1.10) and a statistically significant (*p* = 0.004) reduction in the mean number of working days lost by the parents of children who were treated compared to those who were not (2.58 ± 0.73 vs 1.76 ± 0.76) [42].

In Schaad’s work, which was rated as poor quality according to AMSTAR 2, 8 publications, from 1986 to 2003, in which children with a history of RRIs were monitored for 6 months, comparing OM-85-treated and untreated children, were reviewed. The authors report a statistically significant difference in the incidence of RRIs in the patients treated (32%) compared to the control group (58.2%) [43].

In a subsequent high-quality review [44], including 9 studies published from 1984 until 2003, with a total of 852 children with RRIs, comparing OM-85-treated children (437) and placebo-treated children (415), a statistically significant reduction in the number of acute respiratory infections was observed in the group of children treated compared to the control group [MD (*mean difference*) -1.20; CI 95% -1.75, − 0.66; *p* < 0.0001].

The third and most recent systematic review of 54 studies (4851 children) was published in 2018 and judged to be of moderate quality. The authors, taking data from 44 RCTs, reported a statistically significant association between treatment with OM-85 and a reduction in the frequency of respiratory infections (MD -2.33; 95% CI -2.75, − 1.90; *P* < 0.00001). Furthermore, taking data from 15 RCTs, a statistically significant reduction in fever days in the group treated with OM-85 compared to the control group was reported (MD − 2.91 days; 95% CI -3.75, − 2.07; *p* < 0.00001), along with a reduction in cough days, (MD − 5.26 days; CI 95%-6.41, − 4.12; *P* < 0.00001) and a reduction in days of antibiotic therapy (MD − 4.10 days; CI 95%-4.52, − 3.67; *p* < 0.00001) [45].

The low-quality systematic review by Esposito et al. reports that OM-85 reduced the incidence, prevalence and/or duration of infections in children with a history of RRIs compared to placebo and compared to probiotic therapy [21].

The safety data collected for OM-85 are reassuring, although the drug’s technical data sheet contraindicates its use in the following cases: hypersensitivity to the active substances or any of the excipients, in children under 1 year of age, with autoimmune diseases or acute intestinal infections. Moreover, an interval of 4 weeks between the end of treatment with OM-85 and the start of vaccine administration is recommended [46].

As reported in the datasheet of the product sold in Italy with more than 500 million units of OM-85 prescribed for adults and children, one isolated case of toxic necrotic epidermolysis in a child was reported. The relationship with the use of OM-85 has been estimated as possible, considering that other causes may have contributed to this adverse event (e.g., *Mycoplasma pneumoniae* infection). In some cases, asthma attacks have been observed in predisposed patients after taking drugs containing bacterial extracts; in this case, OM-85 is contraindicated [46].

In general, the frequency of adverse events observed is estimated to be extremely low compared to the high exposure to the product.

In 2018, an AIFA document on safety and efficacy data and claims on the use of bacterial lysates was published (https://www.ema.europa.eu/en/documents/referral/bacterial-lysate-medicines-article-31-referral-notification_en.pdf). The authors of the document were called for the EU to take a stance regarding indications on the use of bacterial lysates. On June 27, 2019 EMA recommended the use of medicines containing bacterial lysate only for the prevention of RRIs, with the exception of pneumonia. The EMA’s recommendation followed a review that concluded the absence of reliable data to show that these medicines are effective in treating respiratory infections or preventing pneumonia and should not be used for these purposes. In the review, the EMA’s *Committee for Medicinal Products for Human Use* (CHMP) looked at the results of clinical trials, data on side effects and advice from a panel of experts on infectious diseases. Although the data were limited, the review found some evidence of the efficacy of these products in preventing RRIs, and the safety profile was in line with that expected for this type of product. The CHMP therefore recommended the use of these medicines for the prevention of RRIs, but pharmaceutical companies must provide additional safety and efficacy data with new clinical studies by 2026.

In conclusion, although the data in the majority of cases suggest the efficacy of OM-85 in the prevention of RRIs, only 2 RCTs, of low to moderate quality, have been conducted in limited numbers of children, and they were produced by one research group only. Consequently, the evidence currently available does not allow us to recommend the routine use of OM-85 for the prevention of RRIs, but it can be recommended in selected populations of children, particularly in children with higher number of RRIs/year, always considering the cost-benefit ratio.

### Vitamins and trace elements

Vitamins are multifunctional compounds belonging to the category of micronutrients. They carry out biological activities that are essential for the completion of enzymatic processes and the health of the human body. Some of them can modulate the functions of the immune system.

Trace elements, which are present in very small quantities in the body, play a fundamental role in the metabolism and proper functioning of the immune system. The increased risk of infection in deficient states has led to the hypothesis that dietary supplements of trace elements can improve the immune response [47]. Zinc, copper and iron are the trace elements involved in the development of the immune response.

At the end of the selection process, 20 full-texts were included, including 5 RCTs [48–52] and 8 observational studies [53–60] of low or very low quality, assessed using the GRADE method, and 7 systematic reviews assessed with the AMSTAR 2 tool, 4 of which were of high quality [61–64], 1 of moderate quality [65], and 2 of very low quality [66, 67].

As regards the efficacy of trace elements in the prevention of RRIs, we have a few low-quality intervention studies available, including 3 RCTs [49, 50, 52], 1 observational study [60], 1 systematic review [62] and 1 meta-analysis [68]. The studies currently available in the literature are burdened by a lack of reproducibility, methodological imprecision, low population size and the heterogeneity of the population studied and of the results obtained, so it is not possible to recommend the use of trace elements in the prevention of RRIs. In response to the outcome about the possible relationship between reduced serum levels of vitamin D/ vitamin A/ vitamin E and increased risk of RRIs in children, 7 observational studies of very low methodological quality and with very heterogeneous results were included [53–56, 58, 59, 69]. The outcomes are different: 3 studies [54, 56, 59] enrolled subjects with RRIs, 1 study [55] selected subjects with AOM, 1 study [53] with recurrent tonsillitis, and, lastly, Shokrollahi’s study assessed subjects with lower respiratory tract infections. The studies by Cayir, Ingham, Zhang and Science show significantly lower vitamin D serum levels in children with RRIs, while the studies by Aydin and Shokrollah show no significant difference in vitamin D serum levels, which are low in both children with RRIs and controls. No studies are currently available in the literature showing that low levels of vitamin A and E create a predisposition for respiratory infections in children, so their use cannot be recommended in the prevention of RRIs.

Two of the intervention studies selected concern the efficacy of vitamin D in the prevention of RRIs, 1 RCT [48] and 1 observational study [57] of low quality, characterised by different outcomes and significant heterogeneity of the populations studied, with non-homogeneous results. The effects of the administration of vitamin D in the prevention of RRIs have also been the subject of systematic reviews and meta-analyses, 5 of which considered eligible [63–67]. Assessment with the AMSTAR 2 tool found 2 of these to be very low quality [66, 67], 2 to be of high quality [63, 64] and 1 to be of moderate quality [65]. Three of them included studies in adults [64, 66, 67]; 1 included only studies carried out in children under the age of 5 and considered the effects of vitamin D supplementation in the prevention of infections in general, including gastrointestinal infections [63]. The majority of them also included patients with asthma, COPD (Chronic Obstructive Pulmonary Disease) or influenza [64–67]. Twenty-five RCTs were included in the most recent meta-analysis [64], only 10 of which were studied in children or adolescents that also included asthma exacerbations or influenza prevention as an outcome*.* The authors concluded the efficacy of vitamin D in preventing infections [adjusted odds ratio (AOR) 0.88, CI 95% 0.81–0.96, heterogeneity *p* < 0.001] with an effect detectable only through daily or weekly administrations but not in a bolus. Furthermore, the effects were greater in subjects with vitamin D values < 25 nmol/l.

The high quality meta-analysis of Yakoob et al. showed no benefit of the administration of vitamin D in the prevention of pneumonia [63]. The authors of the other meta-analysis of moderate quality [65] concluded that there is no evidence to justify the routine use of vitamin D in the prevention of RRIs; however, potential benefits in children with asthma were highlighted. Limited evidences indicate some benefits of vitamin D supplementation in the prevention of recurrent acute otitis media [70].

The reviews assessed as low, moderate and high-quality show different results, with non-homogeneous study populations. It is therefore not possible to recommend the routine use of vitamin D in the prophylaxis of RRIs unless a condition of vitamin D insufficiency or higher risk of low vitamin D levels exist.

As reported in the Italian Consensus on vitamin D in paediatric age published on 2015, the available epidemiological studies show a high prevalence of hypovitaminosis D (above 50%) throughout Italy. Vitamin D status of newborns is influenced by ethnicity, season of birth, and maternal vitamin D status during pregnancy; vitamin D status of children and adolescents is influenced by sun exposure, seasonality, ethnicity, and body mass index. The vitamin D supplementation should be recommended in all infants in the first year of life, independently of the type of feeding. Supplementation should be subsequently individualized in terms of regimen and duration on the basis of the presence of risk factors for vitamin D deficiency. Non-Caucasian ethnicity with dark skin pigmentation, reduced sunlight exposure and/or constant use of sunscreens, international adoption, obesity, inadequate diets (i.e. vegan diet), chronic kidney disease, hepatic failure and/or cholestasis, malabsorption syndromes (i.e. cystic fibrosis, inflammatory bowel diseases, celiac disease at diagnosis, etc.) and chronic therapies (anticonvulsants, systemic glucocorticoids, antiretroviral therapy, systemic antifungals) are the most important risk factors for vitamin D deficiency between 1 and 18 years of age. In the presence of risk factors for vitamin D deficiency the supplementation is recommended according to the dosages reported in the Consensus [71].

The studies currently present in the literature on vitamin C supplementation in the prevention of RRIs, an RCT [51] of very low quality and a systematic review of high methodological quality [61], are burdened by the heterogeneity and a low number of populations studied and the diversity of treatments used, and so do not allow the recommendation of its routine use in the prevention of these episodes. The authors conclude that regular vitamin C supplementation does not reduce the incidence of the common cold in the general population. Although regular supplementation can reduce the duration and severity of episodes, this was not reproducible in the few therapeutic trials performed. Further data are therefore needed.

### Complementary/alternative medicines

Medicines other than official medicine have been given different names over the years: Non-Conventional Medicines, Alternative Medicines, Complementary and Alternative Medicines (CAM), up to the recent proposal of the term Complementary and Integrative Medicines (CIM).

At the end of the selection process, 18 full-texts were included, including 9 systematic reviews assessed using the 16-item AMSTAR questionnaire, and 9 studies assessed with the GRADE method (4 RCTs, 1 randomised open-label study, 2 uncontrolled clinical studies, 1 cohort study and 1 retrospective study).

As regards the effectiveness of homeopathy in reducing the number of RRIs episodes, 2 moderate-quality RCTs [72, 73] and 1 low-quality retrospective observational study [74] were included. The first 2 studies demonstrated no significant effect on reducing the number of episodes; 1 reported effects on symptom severity, appetite and vitality status. The retrospective observational study reporting a reduction in the number of episodes in the group treated with the homoeopathic product is of low quality, both because of the observational nature of the design and because of the absence of a control group treated with placebo. On the other hand, concerning the efficacy of homoeopathy in reducing the use of antibiotics to treat episodes of RRIs, the same 2 moderate-quality RCTs and 1 very low-quality observational study [75] were included; the results of the studies are heterogeneous and, also given their low numbers, it is not possible to provide recommendations regarding the routine use of homoeopathy in this area. Only 2 of the studies included [76, 77], low-quality unblinded RCTs, investigated the efficacy of homeopathy in reducing the intensity and duration of symptoms in episodes of respiratory infection, both reporting a positive result.

Due to the small number of studies currently available on Beta Glucan in the prevention of RRIs episodes, it is not possible to make recommendations in this sense.

In the field of phytotherapy, the effectiveness of *Echinacea* in reducing the number of episodes of RRIs has been assessed; only one non-randomised intervention study [78] is available; it is of very low quality since it lacks a control group, is of low generalisability as it concerns children with otitis or tonsillitis and is imprecise with regard to the number of episodes considered. The Cochrane systematic review [79] included does not show significant efficacy of *Echinacea* preparations in the prevention of common colds. Moreover, it is worth bearing in mind that there is a significant risk of allergic reaction when using *Echinacea* in children under the age of 12. Studies in literature on the use of herbal extracts based on *Pelargonium sidoides* are scarce and of low quality, so there is currently no evidence to support the use of such products in the prevention of RRIs. Regarding the efficacy of *Yupingfen* (a preparation used in Chinese Traditional Medicine) only one study [80], a meta-analysis of moderate quality, was included, as well as for Oscillococcinum only one Cochrane systematic review [81], also of moderate quality, is available. Further studies are therefore needed to provide recommendations.

### Vaccinations

Regarding the role of pneumococcal and influenza vaccinations in the prevention of RRIs, there are few studies currently available in literature; the panel identified only 2 studies, 1 RCT and 1 observational study of low to moderate quality.

In the randomised, double-blind, moderate-quality study by Esposito et al. children with RRIs aged between 6 months and 9 years were given either trivalent inactivated virosomal influenza vaccine (*n* = 64) or placebo (*n* = 63) to assess the number of upper and lower airway infections. The study showed vaccine efficacy in the prevention of upper airway infections of 27% (*p* < 0.0001) and 33% (*p* = 0.03) in the prevention of lower airway infections. Influenza vaccination seems, therefore, to be effective in reducing RRIs in children. Parallel to the significant reduction in infections, an equally significant reduction in the loss of school days (61% efficacy; *p* < 0.0001) and days with fever (23% efficacy; *p* = 0.02) is reported, while the effect on the rate of hospitalisations remains unchanged (1.31 ± 1.33 vs *2.*35 ± 1.59: 44%; *p* < 0*.*0001) [82].

Estrada et al. in a very low-quality retrospective observational study of 72 patients aged 2–25 years, with RRIs, who underwent PCV23 vaccination, assessed the efficacy of vaccination at 1, 3 and 6 months. Clinical response was present in 96% of children with a 50% reduction in episodes or resolution of episodes after 3 months [83].

In conclusion, considering the general safety and benefits of these vaccinations, the panel believes that the general advantages of their use in paediatrics may support their administration, although the strength of the recommendation remains weak due to the limited amount of literature available on RRIs prevention.

### Nasal therapies with hyaluronic acid, thermal waters and resveratrol

To investigate the role of nasal and thermal treatments in the prevention of RRIs, 8 papers were selected, 4 of which were systematic reviews assessed with the AMSTAR II questionnaire [84–87]. The first 3 were of very low quality, while the last was of high quality. The other 4 papers were clinical studies, 3 RCTs [88–90] of low, moderate and low quality respectively, and 1 observational study of moderate quality [91].

Hyaluronic acid is one of the most widely represented components in the extracellular matrix and plays a role in regulating vasomotor tone and mucous gland secretions and in inflammatory processes in the upper and lower airways; consequently, it plays a major role in the effectiveness of mucociliary clearance, which is known to be reduced in patients with rhinitis and chronic rhinosinusitis [92, 93].

As regards the efficacy of nasal therapy with hyaluronic acid in the prevention of RRIs, 5 papers were included, 2 low-quality RCTs [88, 90], 2 very low-quality systematic reviews [84, 85] and 1 more recent good-quality Cochrane review from 2015. The latter assesses the efficacy of nasal irrigation with a saline solution, identifying 3 randomised trials with a total of 544 children. The studies all compared routine care with saline irrigation or other nasal sprays, or otherwise placebo. Most results showed no difference between saline nasal treatment and control. However, the biggest study, conducted in an exclusively paediatric population (aged 6 to 10), showed a significant reduction in nasal secretion score [MD (mean difference) -0.31, CI 95%-0.48, − 0.14], and in nasal obstruction (MD -0.33, CI 95% -0.47, − 0.19) in the group treated with nasal saline. However, an MD − 0.33 on a 4 point symptomatic scale may have minimal clinical significance. The trial also showed a significant reduction in the use of decongestants in the group treated with nasal saline [87].

It has been hypothesised that crenotherapy (inhalation treatment with thermal water) with sodium sulphate-chloride water may modulate the expression of pro-inflammatory cytokines and immunoregulatory and antimicrobial peptides such as *TNF-α* (Tumor Necrosis Factor-α), human β-defensin 2 and calprotectin in the nasal secretions of children with chronic rhinosinusitis. Moreover, the therapeutic activity of thermal water could depend on its mechanical cleansing function and its physical and chemical composition, acting on those alterations of the nasal mucosa typical of inflammatory diseases [94].

As regards the use of crenotherapy in the prevention of RRIs, 2 studies are available, a moderate-quality RCT [89] and a very low-quality systematic review [86]; the studies included showed that, overall, children treated with thermal saline-sulphurous water had a lower number of RRIs than children in the control group, a significant reduction in nasal obstruction, in the degree of turbinate hypertrophy and adenoid hypertrophy, and in the number of neutrophils and bacteria (and biofilms) in the nasal mucosa, as well as a statistically significant improvement in ciliary mucus clearance time.

Resveratrol (R) is a natural non-flavonoid polyphenol belonging to a subclass of stilbenes that has been studied for its possible immunomodulatory action. There is currently only one open-label study, of moderate quality, that has assessed the effect of resveratrol combined with carboxymethyl-β-glucan administered by inhalation in the prevention of RRIs in children. In the group treated, nasal symptoms (obstruction - rhinorrhoea - sneezing) decreased significantly and persistently over time, as did the number of days with cough and fever, the use of medication and the number of school days lost [91].

In conclusion, although the studies available in the literature on the use of nasal therapies with hyaluronic acid, thermal waters and resveratrol in the prevention of RRIs show promising results, they are few in number and of low to moderate quality. For this reason, based on the evidence currently available, the panel does not consider it possible to recommend the use of nasal therapies for the prevention of RRIs.

### Modification of risk factors, antibiotic prophylaxis and adeno/tonsillectomy

Historically, risk factors for the development of RRIs have been divided into modifiable and non-modifiable.

Concerning modifiable factors, the examination of literature led to the inclusion of 15 full texts in the final assessments, 12 of which assessed using the GRADE method and 1 with the AMSTAR 2 tool.

The good-quality review in 2013 by Sauni et al. [95], takes into account 2 environmental factors - damp and mould - and finds that building improvements are associated with a decrease in the number of paediatric visits for acute pathology (mean difference (MD) -0.45; CI 95% -0.76, − 0.14).

Among the modifiable risk factors, diet and food pollution also play an important role; in the studies included, the low-quality study by Calatayud et al. [96] showed an important reduction in episodes of upper respiratory tract infection, in antibiotic use by 87% and in symptomatic treatments by 57% in children with a Mediterranean diet.

The very low-quality observational study by Stølevik et al. assesses the association between prenatal maternal dietary exposure to toxic polychlorinated biphenyls (PCBs) and dioxins and the development of immune-related diseases in children [97].

As regards indoor pollution, 3 low-quality studies are available, 2 of which are observational [98, 99] and 1 cross-sectional [100].

Norbäck et al. [98] conclude that indoor mould, water damage, window condensation, cockroaches and keeping dogs or cats as pets may be risk factors for the common cold while daily cleaning may be a protective factor (OR = 0.89; CI 95% 0.81–0.97).

In the study by Casas et al., the authors conclude that passive exposure to bleach, used for cleaning in the home, can have negative effects on the health of school-age children by increasing the risk of respiratory infections. The high frequency of use of irritating disinfectants for cleaning may be a public health concern [99].

The study by Simoni et al. [100] analysed the relationship between CO_2_ and PM10 concentrations in classrooms and the frequency of respiratory symptoms and diseases (wheezing, night cough and rhinitis).

Only one very low-quality observational study on outdoor pollution was included [101]; the study suggests that prenatal exposure to PM2.5 increases susceptibility to respiratory infections and may influence respiratory morbidity in early childhood.

Regarding the role of smoking, 3 very low-quality studies are available, 1 observational and 2 cross-sectional. The main outcome of the observational study by Marseglia et al. was to assess whether exposure to second-hand smoke altered the immune response and increased the risk of RRIs in children subject to adenoidectomy; children exposed to smoke had more infectious episodes and more courses of antibiotic therapy than children who were not exposed [102].

In an observational study [103] of 64 children, 70.3% of whom were exposed to smoke, Inci et al. showed that, in those exposed, urinary cotinine levels were significantly increased (*p* = 0.011), as was the frequency of acute respiratory infection (*p* = 0.047).

El-Hodhod’s study considered healthy children exposed to smoke and those not exposed, with the former showing a higher frequency of acute bronchitis, dyslipidaemia and significantly higher early lymphocyte apoptosis [104].

Clinicians should always address the emerging problem of the “third-hand smoke” (THS); this term refers to the residue of tobacco smoke that clings to skin, hair clothing, carpets, bags and furniture. Because of their developmental behaviours and their immature immune system infants and children are more prone to the risks related to THS exposure than adults.

Regarding attendance of day-care/preschool, the literature contains data on the association between community placement and environmental exposure to potential pathogens and increased risk of RRIs [105, 106]. However, the panel did not identify studies with the specific outcome of interest.

In conclusion, although some studies support interventions to eliminate certain risk factors in order to prevent RRIs, such as reducing exposure to second-hand smoke, reducing exposure to indoor and outdoor pollutants, such evidence is scarce and obtained from low or very low-quality studies, except for exposure to damp and moulds, for which a good-quality systematic review that supports the elimination of this risk factor is available. However, even on the basis of more general considerations of promoting the general well-being of children, the panel agrees that it is necessary to discourage exposure to second and third-hand smoke and pollutants in general, in addition to improve hand washing as one of the best methods to reduce respiratory infections.

Based current literature review antibiotic prophylaxis and adeno/tonsillectomy are not recommended (Table 2).

## Conclusion

RRIs are a common clinical condition in children, that reduce child and family quality of life and lead to significant medical and social costs. Looking forward to new evidences that could allow the routine use recommendation of drugs for the RRIs prevention, the panel agrees that it is necessary to assesses and contains environmental risk factors, to support the administration of vaccines and to improve the best practices in order to reduce respiratory infections, such as hand washing.

## Supplementary Information

**Additional file 1: Appendix 1**. Search strings. **Appendix 2**. Flowchart of included studies after literature review.

## Data Availability

Not applicable.
