# Supplementary material for: Prevention of recurrent respiratory infections: Inter-society Consensus
Source: Ital J Pediatr. 2021 Oct 25;47:211. doi: 10.1186/s13052-021-01150-0 (PMC8543868; doi:10.1186/s13052-021-01150-0)
Supplement: Supplementary file 1 — Additional file 1: Appendix 1. Search strings. Appendix 2. Flowchart of included studies after literature review. [file 13052_2021_1150_MOESM1_ESM.docx]

**Appendix 1. Search strings**

**Synthetic molecules**

EMBASE

respiratory AND tract AND 'infection'/exp AND (recurrent:ti,ab OR frequent:ti,ab) AND ([infant]/lim OR [child]/lim OR [preschool]/lim OR [school]/lim OR [adolescent]/lim) AND [2009-2019]/py AND ('pidotimod'/exp OR 'pidotimod' OR 'polimod'/exp OR 'polimod' OR 'axil'/exp OR 'axil' OR 'levamisole'/exp OR 'levamisole' OR 'adimod' OR 'isoprinosina' OR 'munostimn' OR 'ribomunyl'/exp OR 'ribomunyl' OR 'lantigen b'/exp OR 'lantigen b' OR 'biostim'/exp OR 'biostim' OR 'ribovac' OR 'adjuvant'/exp OR adjuvant*:ti,ab OR 'immunostimulant*:ti,ab' OR 'immunomodulat*:ti,ab' OR 'immunoadjuvant*:ti,ab')

(recurrent:ti,ab OR frequent:ti,ab) AND respiratory:ti,ab AND infecti*:ti,ab AND [2009-2019]/py AND ([infant]/lim OR [child]/lim OR [preschool]/lim OR [school]/lim OR [adolescent]/lim) AND ('pidotimod'/exp OR 'pidotimod' OR 'polimod'/exp OR 'polimod' OR 'axil'/exp OR 'axil' OR 'levamisole'/exp OR 'levamisole' OR 'adimod' OR 'isoprinosina' OR 'munostimn' OR 'ribomunyl'/exp OR 'ribomunyl' OR 'lantigen b'/exp OR 'lantigen b' OR 'biostim'/exp OR 'biostim' OR 'ribovac' OR 'adjuvant'/exp OR adjuvant*:ti,ab OR 'immunostimulant*:ti,ab' OR 'immunomodulat*:ti,ab' OR 'immunoadjuvant*:ti,ab')

(recurrent:ti,ab OR frequent:ti,ab) AND respiratory:ti,ab AND infecti*:ti,ab AND (child*:ti,ab OR pedia*:ti,ab OR paedia*:ti,ab) AND [2009-2019]/py AND ('pidotimod'/exp OR 'pidotimod' OR 'polimod'/exp OR 'polimod' OR 'axil'/exp OR 'axil' OR 'levamisole'/exp OR 'levamisole' OR 'adimod' OR 'isoprinosina' OR 'munostimn' OR 'ribomunyl'/exp OR 'ribomunyl' OR 'lantigen b'/exp OR 'lantigen b' OR 'biostim'/exp OR 'biostim' OR 'ribovac' OR 'adjuvant'/exp OR adjuvant*:ti,ab OR 'immunostimulant*:ti,ab' OR 'immunomodulat*:ti,ab' OR 'immunoadjuvant*:ti,ab')

PUBMED

(((recurrent OR frequent) AND respiratory AND infecti* AND (child* OR pedia* OR paedia*))) AND (“Pidotimod” OR “Polimod” OR “Adimod” OR “Polimod” OR “Axil” OR “Levamisole” OR “Adimod” OR “Isoprinosina” OR “Munostimn” OR “Ribomunyl” OR “Lantigen B” OR “Biostim” OR “Ribovac” OR adjuvant OR immunologic OR immunostimulant* or immunomodulat* OR immunoadjuvant* OR immunologic adjuvant*) Filters: published in the last 10 years

(((recurrent OR frequent) AND respiratory AND infecti*)) AND (“Pidotimod” OR “Polimod” OR “Adimod” OR “Polimod” OR “Axil” OR “Levamisole” OR “Adimod” OR “Isoprinosina” OR “Munostimn” OR “Ribomunyl” OR “Lantigen B” OR “Biostim” OR “Ribovac” OR adjuvant OR immunologic OR immunostimulant* or immunomodulat* OR immunoadjuvant* OR immunologic adjuvant*) Filters: published in the last 10 years; Child: birth-18 years

(("Respiratory Tract Infections"[Mesh] AND (recurrent[TIAB] OR frequent[TIAB]))) AND (“Pidotimod” OR “Polimod” OR “Adimod” OR “Polimod” OR “Axil” OR “Levamisole” OR “Adimod” OR “Isoprinosina” OR “Munostimn” OR “Ribomunyl” OR “Lantigen B” OR “Biostim” OR “Ribovac” OR adjuvant OR immunologic OR immunostimulant* or immunomodulat* OR immunoadjuvant* OR immunologic adjuvant*) Filters: published in the last 10 years; Child: birth-18 years

**Probiotics, Prebiotics, Symbiotics, Postbiotics**

EMBASE

| respiratory tract infection'/exp AND (recurrent:ti,ab OR frequent:ti,ab) AND ([english]/lim OR [italian]/lim) AND ([infant]/lim OR [child]/lim OR [preschool]/lim OR [school]/lim OR [adolescent]/lim) AND [2009-2019]/py AND ('probiotic agent'/exp OR 'probiotic agent') AND ([embase]/lim OR [pubmed-not-medline]/lim) AND [embase]/lim |
| --- |
| respiratory tract infection'/exp AND (recurrent:ti,ab OR frequent:ti,ab) AND ([infant]/lim OR [child]/lim OR [preschool]/lim OR [school]/lim OR [adolescent]/lim) AND [2009-2019]/py AND ('prebiotic'/exp OR prebiotic) AND ([english]/lim OR [italian]/lim) |
| 'respiratory tract infection'/exp AND (recurrent:ti,ab OR frequent:ti,ab) AND ([infant]/lim OR [child]/lim OR [preschool]/lim OR [school]/lim OR [adolescent]/lim) AND [2009-2019]/py AND ('synbiotic agent'/exp OR 'synbiotic agent') |
| 'respiratory tract infection'/exp AND (recurrent:ti,ab OR frequent:ti,ab) AND ([english]/lim OR [italian]/lim) AND ([infant]/lim OR [child]/lim OR [preschool]/lim OR [school]/lim OR [adolescent]/lim) AND [2009-2019]/py AND 'upper airway microbiome' |
| respiratory tract infection'/exp AND (recurrent:ti,ab OR frequent:ti,ab) AND ([english]/lim OR [italian]/lim) AND ([infant]/lim OR [child]/lim OR [preschool]/lim OR [school]/lim OR [adolescent]/lim) AND [2009-2019]/py AND upper AND ('airway'/exp OR airway) AND ('microbiome'/exp OR microbiome) |
| respiratory tract infection'/exp AND (recurrent:ti,ab OR frequent:ti,ab) AND ([english]/lim OR [italian]/lim) AND ([infant]/lim OR [child]/lim OR [preschool]/lim OR [school]/lim OR [adolescent]/lim) AND [2009-2019]/py AND salivarius |
| 'respiratory tract infection'/exp AND (recurrent:ti,ab OR frequent:ti,ab) AND ([english]/lim OR [italian]/lim) AND ([infant]/lim OR [child]/lim OR [preschool]/lim OR [school]/lim OR [adolescent]/lim) AND [2009-2019]/py AND k12 |
| 'respiratory tract infection'/exp AND (recurrent:ti,ab OR frequent:ti,ab) AND ([english]/lim OR [italian]/lim) AND ([infant]/lim OR [child]/lim OR [preschool]/lim OR [school]/lim OR [adolescent]/lim) AND [2009-2019]/py AND 'fructooligosaccharides' |
| 'respiratory tract infection'/exp AND (recurrent:ti,ab OR frequent:ti,ab) AND ([english]/lim OR [italian]/lim) AND ([infant]/lim OR [child]/lim OR [preschool]/lim OR [school]/lim OR [adolescent]/lim) AND [2009-2019]/py AND gos, AND galactooligosaccharides |
| 'respiratory tract infection'/exp AND (recurrent:ti,ab OR frequent:ti,ab) AND ([english]/lim OR [italian]/lim) AND ([infant]/lim OR [child]/lim OR [preschool]/lim OR [school]/lim OR [adolescent]/lim) AND [2009-2019]/py AND polydextrose, AND pdx |

PUBMED

(recurrent OR frequent) AND respiratory AND infecti* AND (child* OR pedia* OR paedia*) OR (("Respiratory Tract Infections"[Mesh] AND (recurrent[TIAB] OR frequent[TIAB])) AND ("last 10 years"[PDat] AND (infant[MeSH] OR child[MeSH] OR adolescent[MeSH]))) AND ("probiotics"[MeSH Terms] OR probiotic[Text Word]) Filters: published in the last 10 years;

(recurrent OR frequent) AND respiratory AND infecti* AND (child* OR pedia* OR paedia*) OR (("Respiratory Tract Infections"[Mesh] AND (recurrent[TIAB] OR frequent[TIAB])) AND ("last 10 years"[PDat] AND (infant[MeSH] OR child[MeSH] OR adolescent[MeSH]))) AND ("prebiotics"[MeSH Terms] OR prebiotic[Text Word])

 (recurrent OR frequent) AND respiratory AND infecti* AND (child* OR pedia* OR paedia*) OR (("Respiratory Tract Infections"[Mesh] AND (recurrent[TIAB] OR frequent[TIAB])) AND ("last 10 years"[PDat] AND (infant[MeSH] OR child[MeSH] OR adolescent[MeSH]))) AND (FOS[All Fields] AND fructooligosaccharides[All Fields] AND ("Glob Surg"[Journal] OR "gos"[All Fields]) AND galactooligosaccharides[All Fields] OR (("polydextrose"[Supplementary Concept] AND "polydextrose"[All Fields])) AND PDX[All Fields]) Filters: published in the last 10 years; Field: Title/Abstract

(recurrent OR frequent) AND respiratory AND infecti* AND (child* OR pedia* OR paedia*) OR (("Respiratory Tract Infections"[Mesh] AND (recurrent[TIAB] OR frequent[TIAB])) AND ("last 10 years"[PDat] AND (infant[MeSH] OR child[MeSH] OR adolescent[MeSH]))) AND ("streptococcus salivarius"[MeSH Terms] OR ("streptococcus"[All Fields] AND "salivarius"[All Fields]) OR "streptococcus salivarius"[All Fields]) AND "streptococcus oralis"[MeSH Terms] OR ("streptococcus salivarius"[MeSH Terms] OR ("streptococcus"[All Fields] AND "salivarius"[All Fields]) OR "streptococcus salivarius"[All Fields]) AND K12[All Fields]

(recurrent OR frequent) AND respiratory AND infecti* AND (child* OR pedia* OR paedia*) OR (("Respiratory Tract Infections"[Mesh] AND (recurrent[TIAB] OR frequent[TIAB])) AND ("last 10 years"[PDat] AND (infant[MeSH] OR child[MeSH] OR adolescent[MeSH]))) AND (Upper[All Fields] AND airway[All Fields] AND ("microbiota"[MeSH Terms] OR "microbiota"[All Fields] OR "microbiome"[All Fields]) Filters: published in the last 10 years; Field: Title/Abstract

 (recurrent OR frequent) AND respiratory AND infecti* AND (child* OR pedia* OR paedia*) OR (("Respiratory Tract Infections"[Mesh] AND (recurrent[TIAB] OR frequent[TIAB])) AND ("2009/03/11"[PDat] "2019/03/08"[PDat] AND (infant[MeSH] OR child[MeSH] OR adolescent[MeSH]))) AND (synbiotics[MeSH Terms] OR symbiotic)

(recurrent OR frequent) AND respiratory AND infecti* AND (child* OR pedia* OR paedia*) OR (("Respiratory Tract Infections"[Mesh] AND (recurrent[TIAB] OR frequent[TIAB])) AND ("last 10 years"[PDat] AND (infant[MeSH] OR child[MeSH] OR adolescent[MeSH]))) AND (prebiotics[MeSH Terms] OR prebiotic) Filters: published in the last 10 years; Field: Title/Abstract

**Lysates and Bacterial Extracts**

EMBASE

(recurrent:ti,ab OR frequent:ti,ab) AND respiratory:ti,ab AND infecti*:ti,ab AND (child*:ti,ab OR pedia*:ti,ab OR paedia*:ti,ab) AND ('broncho-vaxom' OR 'broncho vaxom' OR 'bronchovaxom' OR 'broncho-munal' OR 'broncho munal' OR 'bronchomunal' OR 'imocur' OR 'om-85' OR 'om 85' OR 'om-85 bv' OR 'om85' OR 'pulmonarom' OR 'pulmonar om' OR 'pulmonar-om' OR 'bactek' OR 'sl-04' OR 'ismigen' OR 'immubron' OR 'vacunace' OR 'bacterial lysate' OR 'irs 19' OR 'irs-19' OR 'irs19' OR 'lw 50020' OR 'lw50020' OR 'imudon' OR 'ru41740' OR 'ru-41740' OR 'ru 41740' OR 'immuncytal' OR 'lantigen b' OR 'biomunil' OR 'buccalin' OR 'luivac' OR 'ommunal' OR 'paspat' OR 'pir 05' OR 'pir-05' OR 'pir05' OR 'polyvaccinum' OR 'provax' OR 'respivax') AND [2009-2019]/py

(recurrent:ti,ab OR frequent:ti,ab) AND respiratory:ti,ab AND infecti*:ti,ab AND ('broncho-vaxom' OR 'broncho vaxom' OR 'bronchovaxom' OR 'broncho-munal' OR 'broncho munal' OR 'bronchomunal' OR 'imocur' OR 'om-85' OR 'om 85' OR 'om-85 bv' OR 'om85' OR 'pulmonarom' OR 'pulmonar om' OR 'pulmonar-om' OR 'bactek' OR 'sl-04' OR 'ismigen' OR 'immubron' OR 'vacunace' OR 'bacterial lysate' OR 'irs 19' OR 'irs-19' OR 'irs19' OR 'lw 50020' OR 'lw50020' OR 'imudon' OR 'ru41740' OR 'ru-41740' OR 'ru 41740' OR 'immuncytal' OR 'lantigen b' OR 'biomunil' OR 'buccalin' OR 'luivac' OR 'ommunal' OR 'paspat' OR 'pir 05' OR 'pir-05' OR 'pir05' OR 'polyvaccinum' OR 'provax' OR 'respivax') AND ([infant]/lim OR [child]/lim OR [preschool]/lim OR [school]/lim OR [adolescent]/lim) AND [2009-2019]/py

('respiratory tract infection'/exp OR 'respiratory tract infection') AND ('broncho-vaxom' OR 'broncho vaxom' OR 'bronchovaxom' OR 'broncho-munal' OR 'broncho munal' OR 'bronchomunal' OR 'imocur' OR 'om-85' OR 'om 85' OR 'om-85 bv' OR 'om85' OR 'pulmonarom' OR 'pulmonar om' OR 'pulmonar-om' OR 'bactek' OR 'sl-04' OR 'ismigen' OR 'immubron' OR 'vacunace' OR 'bacterial lysate' OR 'irs 19' OR 'irs-19' OR 'irs19' OR 'lw 50020' OR 'lw50020' OR 'imudon' OR 'ru41740' OR 'ru-41740' OR 'ru 41740' OR 'immuncytal' OR 'lantigen b' OR 'biomunil' OR 'buccalin' OR 'luivac' OR 'ommunal' OR 'paspat' OR 'pir 05' OR 'pir-05' OR 'pir05' OR 'polyvaccinum' OR 'provax' OR 'respivax') AND ([infant]/lim OR [child]/lim OR [preschool]/lim OR [school]/lim OR [adolescent]/lim) AND [2009-2019]/py

PUBMED

((“Broncho-Vaxom” OR “Broncho Vaxom” OR “bronchovaxom” OR “Broncho-Munal” OR “Broncho Munal” OR “bronchomunal” OR “Imocur” OR “OM-85” OR “OM 85” OR “OM-85 BV” OR “OM85” OR “PulmonarOM” OR “Pulmonar OM” OR “Pulmonar-OM” OR “Respivax” OR “Bactek” OR “SL-04” OR “Ismigen” OR “Immubron” OR “Vacunace” OR “Bacterial lysate” OR “IRS 19” OR “IRS-19” OR “IRS19” OR “Paspat” OR “LW 50020” OR “LW50020” OR “Luivac” OR “Imudon” OR “ru41740” OR “ru-41740” OR “ru 41740” OR “Immuncytal” OR “Lantigen B” OR “Biomunil” OR “Buccalin” OR “Luivac” OR “Ommunal” OR “Paspat” OR “pir 05” OR “pir-05” OR “PIR05” OR “Polyvaccinum” OR “Provax” OR “Respivax”)) AND ((recurrent OR frequent) AND respiratory AND infecti* AND (child* OR pedia* OR paedia*)) Sort by: Best Match Filters: published in the last 10 years

(("Respiratory Tract Infections"[Mesh] AND (recurrent[TIAB] OR frequent[TIAB]))) AND (((“Broncho-Vaxom” OR “Broncho Vaxom” OR “bronchovaxom” OR “Broncho-Munal” OR “Broncho Munal” OR “bronchomunal” OR “Imocur” OR “OM-85” OR “OM 85” OR “OM-85 BV” OR “OM85” OR “PulmonarOM” OR “Pulmonar OM” OR “Pulmonar-OM” OR “Respivax” OR “Bactek” OR “SL-04” OR “Ismigen” OR “Immubron” OR “Vacunace” OR “Bacterial lysate” OR “IRS 19” OR “IRS-19” OR “IRS19” OR “Paspat” OR “LW 50020” OR “LW50020” OR “Luivac” OR “Imudon” OR “ru41740” OR “ru-41740” OR “ru 41740” OR “Immuncytal” OR “Lantigen B” OR “Biomunil” OR “Buccalin” OR “Luivac” OR “Ommunal” OR “Paspat” OR “pir 05” OR “pir-05” OR “PIR05” OR “Polyvaccinum” OR “Provax” OR “Respivax”)) AND ("last 10 years"[PDat] AND (infant[MeSH] OR child[MeSH] OR adolescent[MeSH]))) Sort by: Best Match Filters: published in the last 10 years

(("Respiratory Tract Infections"[Mesh] AND (recurrent[TIAB] OR frequent[TIAB]))) AND (((“Broncho-Vaxom” OR “Broncho Vaxom” OR “bronchovaxom” OR “Broncho-Munal” OR “Broncho Munal” OR “bronchomunal” OR “Imocur” OR “OM-85” OR “OM 85” OR “OM-85 BV” OR “OM85” OR “PulmonarOM” OR “Pulmonar OM” OR “Pulmonar-OM” OR “Respivax” OR “Bactek” OR “SL-04” OR “Ismigen” OR “Immubron” OR “Vacunace” OR “Bacterial lysate” OR “IRS 19” OR “IRS-19” OR “IRS19” OR “Paspat” OR “LW 50020” OR “LW50020” OR “Luivac” OR “Imudon” OR “ru41740” OR “ru-41740” OR “ru 41740” OR “Immuncytal” OR “Lantigen B” OR “Biomunil” OR “Buccalin” OR “Luivac” OR “Ommunal” OR “Paspat” OR “pir 05” OR “pir-05” OR “PIR05” OR “Polyvaccinum” OR “Provax” OR “Respivax”)) AND ("last 10 years"[PDat] AND (infant[MeSH] OR child[MeSH] OR adolescent[MeSH]))) Sort by: Best Match Filters: published in the last 10 years; Child: birth-18 years

**Vitamins and Trace Elements**

EMBASE

'respiratory tract infection'/exp AND (recurrent:ti,ab OR frequent:ti,ab) AND ([infant]/lim OR [child]/lim OR [preschool]/lim OR [school]/lim OR [adolescent]/lim) AND [2009-2019]/py AND ('iron'/exp OR 'zinc'/exp OR 'copper'/exp OR 'manganese'/exp OR 'iodine'/exp OR 'trace element'/exp)

'respiratory tract infection'/exp AND (recurrent:ti,ab OR frequent:ti,ab) AND ([infant]/lim OR [child]/lim OR [preschool]/lim OR [school]/lim OR [adolescent]/lim) AND [2009-2019]/py AND ('vitamin d'/exp OR 'vitamin d' OR 'calcitriol' OR 'ergocalciferols'/exp OR '1 alpha, 25 dihydroxy 20 epi vitamin d3' OR '25 hydroxyvitamin d' OR '25 hydroxy*' OR 'colecalciferol derivative' OR 'colecalciferol'/exp OR 'colecalc*')

'respiratory tract infection'/exp AND (recurrent:ti,ab OR frequent:ti,ab) AND ([infant]/lim OR [child]/lim OR [preschool]/lim OR [school]/lim OR [adolescent]/lim) AND [2009-2019]/py AND ('vitamin a'/exp OR 'retinol' OR 'retinoic acid' OR 'retinoid')

'respiratory tract infection'/exp AND (recurrent:ti,ab OR frequent:ti,ab) AND ([infant]/lim OR [child]/lim OR [preschool]/lim OR [school]/lim OR [adolescent]/lim) AND [2009-2019]/py AND ('vitamin b1'/exp OR 'thiamine')

'respiratory tract infection'/exp AND (recurrent:ti,ab OR frequent:ti,ab) AND ([infant]/lim OR [child]/lim OR [preschool]/lim OR [school]/lim OR [adolescent]/lim) AND [2009-2019]/py AND 'pyridoxine derivative'/exp

'respiratory tract infection'/exp AND (recurrent:ti,ab OR frequent:ti,ab) AND ([infant]/lim OR [child]/lim OR [preschool]/lim OR [school]/lim OR [adolescent]/lim) AND [2009-2019]/py AND ('vitamin b2'/exp OR 'riboflavin')

'respiratory tract infection'/exp AND (recurrent:ti,ab OR frequent:ti,ab) AND ([infant]/lim OR [child]/lim OR [preschool]/lim OR [school]/lim OR [adolescent]/lim) AND [2009-2019]/py AND ('vitamin b12'/exp OR 'cyanocobalamin')

| 'respiratory tract infection'/exp AND (recurrent:ti,ab OR frequent:ti,ab) AND ([infant]/lim OR [child]/lim OR [preschool]/lim OR [school]/lim OR [adolescent]/lim) AND [2009-2019]/py AND ('ascorbic acid'/exp OR 'vitamin c') |
| --- |
| 'respiratory tract infection'/exp AND (recurrent:ti,ab OR frequent:ti,ab) AND ([infant]/lim OR [child]/lim OR [preschool]/lim OR [school]/lim OR [adolescent]/lim) AND [2009-2019]/py AND ('alpha tocopherol'/exp OR 'vitamin e') |
| 'respiratory tract infection'/exp AND (recurrent:ti,ab OR frequent:ti,ab) AND ([infant]/lim OR [child]/lim OR [preschool]/lim OR [school]/lim OR [adolescent]/lim) AND [2009-2019]/py AND ('pantothenic acid'/exp OR 'vitamin b5) |
| 'respiratory tract infection'/exp AND (recurrent:ti,ab OR frequent:ti,ab) AND ([infant]/lim OR [child]/lim OR [preschool]/lim OR [school]/lim OR [adolescent]/lim) AND [2009-2019]/py AND 'biotin derivative'/exp |
| 'respiratory tract infection'/exp AND (recurrent:ti,ab OR frequent:ti,ab) AND ([infant]/lim OR [child]/lim OR [preschool]/lim OR [school]/lim OR [adolescent]/lim) AND [2009-2019]/py AND 'vitamin b7' |

PUBMED

(recurrent OR frequent) AND respiratory AND infecti* AND (child* OR pedia* OR paedia*) OR (("Respiratory Tract Infections"[Mesh] AND (recurrent[TIAB] OR frequent[TIAB])) AND ("last 10 years"[PDat] AND (infant[MeSH] OR child[MeSH]

OR adolescent[MeSH]))) AND (Iron OR Zinc OR Copper OR Manganese OR Iodine) Filters: published in the last 10 years

(recurrent OR frequent) AND respiratory AND infecti* AND (child* OR pedia* OR paedia*) OR (("Respiratory Tract Infections"[Mesh] AND (recurrent[TIAB] OR frequent[TIAB])) AND ("last 10 years"[PDat] AND (infant[MeSH] OR child[MeSH] OR adolescent[MeSH]))) AND (“Iodine” [MeSH Terms])Schema: all Filters: published in the last 10 years

(recurrent OR frequent) AND respiratory AND infecti* AND (child* OR pedia* OR paedia*) OR (("Respiratory Tract Infections"[Mesh] AND (recurrent[TIAB] OR frequent[TIAB])) AND ("last 10 years"[PDat] AND (infant[MeSH] OR child[MeSH] OR adolescent[MeSH]))) AND (“Iodine” [MeSH Terms])Filters: published in the last 10 years

(recurrent OR frequent) AND respiratory AND infecti* AND (child* OR pedia* OR paedia*) OR (("Respiratory Tract Infections"[Mesh] AND (recurrent[TIAB] OR frequent[TIAB])) AND ("last 10 years"[PDat] AND (infant[MeSH] OR child[MeSH] OR adolescent[MeSH]))) AND (“Manganese” [MeSH Terms]) Schema: all Filters: published in the last 10 years

(recurrent OR frequent) AND respiratory AND infecti* AND (child* OR pedia* OR paedia*) OR (("Respiratory Tract Infections"[Mesh] AND (recurrent[TIAB] OR frequent[TIAB])) AND ("last 10 years"[PDat] AND (infant[MeSH] OR child[MeSH] OR adolescent[MeSH]))) AND (“Manganese” [MeSH Terms]) Filters: published in the last 10 years

(recurrent OR frequent) AND respiratory AND infecti* AND (child* OR pedia* OR paedia*) OR (("Respiratory Tract Infections"[Mesh] AND (recurrent[TIAB] OR frequent[TIAB])) AND ("last 10 years"[PDat] AND (infant[MeSH] OR child[MeSH] OR adolescent[MeSH]))) AND (“copper” [MeSH Terms])Filters: published in the last 10 years

(recurrent OR frequent) AND respiratory AND infecti* AND (child* OR pedia* OR paedia*) OR (("Respiratory Tract Infections"[Mesh] AND (recurrent[TIAB] OR frequent[TIAB])) AND ("last 10 years"[PDat] AND (infant[MeSH] OR child[MeSH] OR adolescent[MeSH]))) AND (“Zinc” [MeSH Terms]) Filters: published in the last 10 years

(recurrent OR frequent) AND respiratory AND infecti* AND (child* OR pedia* OR paedia*) OR (("Respiratory Tract Infections"[Mesh] AND (recurrent[TIAB] OR frequent[TIAB])) AND ("last 10 years"[PDat] AND (infant[MeSH] OR child[MeSH] OR adolescent[MeSH]))) AND (“Iron” [MeSH Terms] )Filters: published in the last 10 years

(recurrent OR frequent) AND respiratory AND infecti* AND (child* OR pedia* OR paedia*) OR (("Respiratory Tract Infections"[Mesh] AND (recurrent[TIAB] OR frequent[TIAB])) AND ("last 10 years"[PDat] AND (infant[MeSH] OR child[MeSH] OR adolescent[MeSH]))) AND (“Iron”[MeSH Terms] OR “Zinc”[MeSH Terms] OR “Copper”[MeSH Terms] OR “Manganese”[MeSH Terms] OR “Iodine”[MeSH Terms] OR "trace element"[MeSH Terms] OR "trace elements"[MeSH Terms]) Filters: published in the last 10 years

(recurrent OR frequent) AND respiratory AND infecti* AND (child* OR pedia* OR paedia*) OR (("Respiratory Tract Infections"[Mesh] AND (recurrent[TIAB] OR frequent[TIAB])) AND ("last 10 years"[PDat] AND (infant[MeSH] OR child[MeSH] OR adolescent[MeSH]))) AND ("Vitamin B7” [MeSH Terms])Schema: all Filters: published in the last 10 years

(recurrent OR frequent) AND respiratory AND infecti* AND (child* OR pedia* OR paedia*) OR (("Respiratory Tract Infections"[Mesh] AND (recurrent[TIAB] OR frequent[TIAB])) AND ("last 10 years"[PDat] AND (infant[MeSH] OR child[MeSH] OR adolescent[MeSH]))) AND ("Vitamin B7” [MeSH Terms])Filters: published in the last 10 years

(recurrent OR frequent) AND respiratory AND infecti* AND (child* OR pedia* OR paedia*) OR (("Respiratory Tract Infections"[Mesh] AND (recurrent[TIAB] OR frequent[TIAB])) AND ("last 10 years"[PDat] AND (infant[MeSH] OR child[MeSH] OR adolescent[MeSH]))) AND ("Vitamin H” [MeSH Terms] )Schema: all Filters: published in the last 10 years

(recurrent OR frequent) AND respiratory AND infecti* AND (child* OR pedia* OR paedia*) OR (("Respiratory Tract Infections"[Mesh] AND (recurrent[TIAB] OR frequent[TIAB])) AND ("last 10 years"[PDat] AND (infant[MeSH] OR child[MeSH] OR adolescent[MeSH]))) AND ("Vitamin H” [MeSH Terms] )Filters: published in the last 10 years

recurrent OR frequent) AND respiratory AND infecti* AND (child* OR pedia* OR paedia*) OR (("Respiratory Tract Infections"[Mesh] AND (recurrent[TIAB] OR frequent[TIAB])) AND ("last 10 years"[PDat] AND (infant[MeSH] OR child[MeSH] OR adolescent[MeSH]))) AND ("Vitamin B5” [MeSH Terms])Schema: all Filters: published in the last 10 years

recurrent OR frequent) AND respiratory AND infecti* AND (child* OR pedia* OR paedia*) OR (("Respiratory Tract Infections"[Mesh] AND (recurrent[TIAB] OR frequent[TIAB])) AND ("last 10 years"[PDat] AND (infant[MeSH] OR child[MeSH] OR adolescent[MeSH]))) AND ("Vitamin B5” [MeSH Terms])Filters: published in the last 10 years

(recurrent OR frequent) AND respiratory AND infecti* AND (child* OR pedia* OR paedia*) OR (("Respiratory Tract Infections"[Mesh] AND (recurrent[TIAB] OR frequent[TIAB])) AND ("last 10 years"[PDat] AND (infant[MeSH] OR child[MeSH] OR adolescent[MeSH]))) AND ("Vitamin E” [MeSH Terms])Filters: published in the last 10 years

(recurrent OR frequent) AND respiratory AND infecti* AND (child* OR pedia* OR paedia*) OR (("Respiratory Tract Infections"[Mesh] AND (recurrent[TIAB] OR frequent[TIAB])) AND ("last 10 years"[PDat] AND (infant[MeSH] OR child[MeSH] OR adolescent[MeSH]))) AND ("Vitamin C” [MeSH Terms] )Schema: all Filters: published in the last 10 years

(recurrent OR frequent) AND respiratory AND infecti* AND (child* OR pedia* OR paedia*) OR (("Respiratory Tract Infections"[Mesh] AND (recurrent[TIAB] OR frequent[TIAB])) AND ("last 10 years"[PDat] AND (infant[MeSH] OR child[MeSH] OR adolescent[MeSH]))) AND ("Vitamin C” [MeSH Terms] )Filters: published in the last 10 years

(recurrent OR frequent) AND respiratory AND infecti* AND (child* OR pedia* OR paedia*) OR (("Respiratory Tract Infections"[Mesh] AND (recurrent[TIAB] OR frequent[TIAB])) AND ("last 10 years"[PDat] AND (infant[MeSH] OR child[MeSH] OR adolescent[MeSH]))) AND ("Vitamin B12” [MeSH Terms]) Schema: all Filters: published in the last 10 years

(recurrent OR frequent) AND respiratory AND infecti* AND (child* OR pedia* OR paedia*) OR (("Respiratory Tract Infections"[Mesh] AND (recurrent[TIAB] OR frequent[TIAB])) AND ("last 10 years"[PDat] AND (infant[MeSH] OR child[MeSH] OR adolescent[MeSH]))) AND ("Vitamin B12” [MeSH Terms]) Filters: published in the last 10 years

(recurrent OR frequent) AND respiratory AND infecti* AND (child* OR pedia* OR paedia*) OR (("Respiratory Tract Infections"[Mesh] AND (recurrent[TIAB] OR frequent[TIAB])) AND ("last 10 years"[PDat] AND (infant[MeSH] OR child[MeSH] OR adolescent[MeSH]))) AND ("Vitamin B6” [MeSH Terms] ) Schema: all Filters: published in the last 10 years

(recurrent OR frequent) AND respiratory AND infecti* AND (child* OR pedia* OR paedia*) OR (("Respiratory Tract Infections"[Mesh] AND (recurrent[TIAB] OR frequent[TIAB])) AND ("last 10 years"[PDat] AND (infant[MeSH] OR child[MeSH] OR adolescent[MeSH]))) AND ("Vitamin B6” [MeSH Terms] ) Filters: published in the last 10 years

(recurrent OR frequent) AND respiratory AND infecti* AND (child* OR pedia* OR paedia*) OR (("Respiratory Tract Infections"[Mesh] AND (recurrent[TIAB] OR frequent[TIAB])) AND ("last 10 years"[PDat] AND (infant[MeSH] OR child[MeSH] OR adolescent[MeSH]))) AND ("Vitamin B6” [MeSH Terms])Schema: all Filters: published in the last 10 years

(recurrent OR frequent) AND respiratory AND infecti* AND (child* OR pedia* OR paedia*) OR (("Respiratory Tract Infections"[Mesh] AND (recurrent[TIAB] OR frequent[TIAB])) AND ("last 10 years"[PDat] AND (infant[MeSH] OR child[MeSH] OR adolescent[MeSH]))) AND ("Vitamin B6” [MeSH Terms])Filters: published in the last 10 years

 (recurrent OR frequent) AND respiratory AND infecti* AND (child* OR pedia* OR paedia*) OR (("Respiratory Tract Infections"[Mesh] AND (recurrent[TIAB] OR frequent[TIAB])) AND ("last 10 years"[PDat] AND (infant[MeSH] OR child[MeSH] OR adolescent[MeSH]))) AND ("Vitamin B2” [MeSH Terms] ) Schema: all Filters: published in the last 10 years

(recurrent OR frequent) AND respiratory AND infecti* AND (child* OR pedia* OR paedia*) OR (("Respiratory Tract Infections"[Mesh] AND (recurrent[TIAB] OR frequent[TIAB])) AND ("last 10 years"[PDat] AND (infant[MeSH] OR child[MeSH] OR adolescent[MeSH]))) AND ("Vitamin B2” [MeSH Terms] ) Filters: published in the last 10 years

(recurrent OR frequent) AND respiratory AND infecti* AND (child* OR pedia* OR paedia*) OR (("Respiratory Tract Infections"[Mesh] AND (recurrent[TIAB] OR frequent[TIAB])) AND ("last 10 years"[PDat] AND (infant[MeSH] OR child[MeSH] OR adolescent[MeSH]))) AND (“Vitamin B1” [MeSH Terms])Schema: all Filters: published in the last 10 years

(recurrent OR frequent) AND respiratory AND infecti* AND (child* OR pedia* OR paedia*) OR (("Respiratory Tract Infections"[Mesh] AND (recurrent[TIAB] OR frequent[TIAB])) AND ("last 10 years"[PDat] AND (infant[MeSH] OR child[MeSH] OR adolescent[MeSH]))) AND (“Vitamin B1” [MeSH Terms])Filters: published in the last 10 years

(recurrent OR frequent) AND respiratory AND infecti* AND (child* OR pedia* OR paedia*) OR (("Respiratory Tract Infections"[Mesh] AND (recurrent[TIAB] OR frequent[TIAB])) AND ("last 10 years"[PDat] AND (infant[MeSH] OR child[MeSH] OR adolescent[MeSH]))) AND (“Vitamin D”[MeSH Terms] OR "calcitriol"[MeSH Terms] OR "ergocalciferols"[MeSH Terms] OR "1 alpha, 25 dihydroxy 20 epi vitamin d3" OR "25 hydroxyvitamin D"[MeSH Terms] OR "25 hydroxy*" OR "colecalciferol derivative"[MeSH Terms]) Filters: published in the last 10 years

(recurrent OR frequent) AND respiratory AND infecti* AND (child* OR pedia* OR paedia*) OR (("Respiratory Tract Infections"[Mesh] AND (recurrent[TIAB] OR frequent[TIAB])) AND ("last 10 years"[PDat] AND (infant[MeSH] OR child[MeSH] OR adolescent[MeSH]))) AND ("vitamin A"[MeSH] OR "retinol"[MeSH] OR "retinoic acid"[MeSH] OR "retinoid"[MeSH]) Filters: published in the last 10 years

(recurrent OR frequent) AND respiratory AND infecti* AND (child* OR pedia* OR paedia*) OR (("Respiratory Tract Infections"[Mesh] AND (recurrent[TIAB] OR frequent[TIAB])) AND ("last 10 years"[PDat] AND (infant[MeSH] OR child[MeSH] OR adolescent[MeSH]))) AND ("Vitamin D"[MeSH] OR "vitamin D2"[MeSH] OR "vitamin D3"[MeSH] OR "cholecalciferol"[MeSH] OR "ergocalciferol"[MeSH] OR "Vitamin A"[MeSH] OR "Vitamin B1"[MeSH] OR "thiamine"[MeSH] OR "Vitamin B2"[MeSH] OR "riboflavin"[MeSH] OR "Vitamin B6"[MeSH] OR "pyridoxine"[MeSH] OR "Vitamin B12"[MeSH] OR "cobalamin"[MeSH] OR "Vitamin C"[MeSH] OR "ascorbic acid"[MeSH] OR "Vitamin E"[MeSH] OR "Pantothenic acid"[MeSH] OR "Vitamin B5"[MeSH] OR "Biotin"[MeSH] OR "Vitamin H"[MeSH] OR "Vitamin B7"[MeSH]) Filters: published in the last 10 years

recurrent OR frequent) AND respiratory AND infecti* AND (child* OR pedia* OR paedia*) OR (("Respiratory Tract Infections"[Mesh] AND (recurrent[TIAB] OR frequent[TIAB])) AND ("last 10 years"[PDat] AND (infant[MeSH] OR child[MeSH] OR adolescent[MeSH]))) AND ("Vitamin D"[MeSH] OR "vitamin D2"[MeSH] OR "vitamin D3"[MeSH] OR "cholecalciferol"[MeSH] OR "ergocalciferol"[MeSH] OR "Vitamin A"[MeSH] OR "Vitamin B1"[MeSH] OR "thiamine"[MeSH] OR "Vitamin B2"[MeSH] OR "riboflavin"[MeSH] OR "Vitamin B6"[MeSH] OR "pyridoxine"[MeSH] OR "Vitamin B12"[MeSH] OR "cobalamin"[MeSH] OR "Vitamin C"[MeSH] OR "ascorbic acid"[MeSH] OR "Vitamin E"[MeSH] OR "Pantothenic acid"[MeSH] OR "Vitamin B5"[MeSH] OR "Biotin"[MeSH] OR "Vitamin H"[MeSH] OR "Vitamin B7"[MeS])

**Complementary/Alternative Medicines**

EMBASE

'respiratory tract infection'/exp AND (recurrent:ti,ab OR frequent:ti,ab) AND ([english]/lim OR [italian]/lim) AND ([infant]/lim OR [child]/lim OR [preschool]/lim OR [school]/lim OR [adolescent]/lim) AND [2009-2019]/py AND (‘complementary therapies’ OR halotherapy OR thermal OR homeopath* OR phytotherapy OR homotoxicology)

(recurrent:ti,ab OR frequent:ti,ab) AND respiratory:ti,ab AND infecti*:ti,ab AND ([english]/lim OR [italian]/lim) AND [2009-2019]/py AND ([infant]/lim OR [child]/lim OR [preschool]/lim OR [school]/lim OR [adolescent]/lim) AND (‘complementary therapies’ OR halotherapy OR thermal OR homeopath* OR phytotherapy OR homotoxicology)

(recurrent:ti,ab OR frequent:ti,ab) AND respiratory:ti,ab AND infecti*:ti,ab AND (child*:ti,ab OR pedia*:ti,ab OR paedia*:ti,ab) AND ([english]/lim OR [italian]/lim) AND [2009-2019]/py AND (‘complementary therapies’ OR halotherapy OR thermal OR homeopath* OR phytotherapy OR homotoxicology)

PUBMED

((recurrent OR frequent) AND respiratory AND infecti* AND (child* OR pedia* OR paedia*)) OR (("Respiratory Tract Infections"[Mesh] AND (recurrent[TIAB] OR frequent[TIAB])) AND ("last 10 years"[PDat] AND (infant[MeSH] OR child[MeSH] OR adolescent[MeSH])))) AND (“complementary therapies” OR halotherapy OR thermal OR homeopath* OR phytotherapy OR homotoxicology)

**Vaccinations**

EMBASE

('respiratory tract infection'/exp OR 'respiratory tract infection') AND (recurrent:ti,ab OR frequent:ti,ab) AND ('pneumococcal' OR 'influenza'/exp OR 'influenza' OR 'vaccination*' OR 'vaccin*' OR 'immunisation'/exp OR 'immunisation' OR 'immunization'/exp OR 'immunization' OR 'pneumovax'/exp OR 'pneumovax' OR 'pnu-imune'/exp OR 'pnu-imune' OR 'prevenar'/exp OR 'prevenar' OR 'pcv7' OR '7-valent' OR 'seven valent' OR 'phid-cv10' OR 'phid-cv'/exp OR 'phid-cv' OR 'pcv-13'/exp OR 'pcv-13' OR '13-valent' OR 'thirteen valent' OR 'flu'/exp OR 'flu' OR 'lav vaccine') AND ([infant]/lim OR [child]/lim OR [preschool]/lim OR [school]/lim OR [adolescent]/lim) AND [2009-2019]/py

(recurrent:ti,ab OR frequent:ti,ab) AND respiratory:ti,ab AND infecti*:ti,ab AND ('pneumococcal' OR 'influenza' OR 'vaccination*' OR 'vaccin*' OR 'immunisation' OR 'immunization' OR 'pneumovax' OR 'pnu-imune' OR 'prevenar' OR 'pcv7' OR '7-valent' OR 'seven valent' OR 'phid-cv10' OR 'phid-cv' OR 'pcv-13' OR '13-valent' OR 'thirteen valent' OR 'flu' OR 'lav vaccine') AND ([infant]/lim OR [child]/lim OR [preschool]/lim OR [school]/lim OR [adolescent]/lim) AND [2009-2019]/py

(recurrent:ti,ab OR frequent:ti,ab) AND respiratory:ti,ab AND infecti*:ti,ab AND (child*:ti,ab OR pedia*:ti,ab OR paedia*:ti,ab) AND ('pneumococcal' OR 'influenza' OR 'vaccination*' OR 'vaccin*' OR 'immunisation' OR 'immunization' OR 'pneumovax' OR 'pnu-imune' OR 'prevenar' OR 'pcv7' OR '7-valent' OR 'seven valent' OR 'phid-cv10' OR 'phid-cv' OR 'pcv-13' OR '13-valent' OR 'thirteen valent' OR 'flu' OR 'lav vaccine') AND [2009-2019]/py

PUBMED

(((recurrent OR frequent) AND respiratory AND infecti* AND (child* OR pedia* OR paedia*))) AND (“Pneumococcal” OR “Influenza” OR “Vaccination*” OR “vaccin*” OR “Immunisation” OR “immunization” OR “Pneumovax” OR “Pnu-imune” OR “Prevenar” OR “PCV7” OR “7-valent” OR “seven valent” OR “PHiD-CV10” OR “PhiD-CV” OR “PCV-13” OR “13-valent” OR “thirteen valent” OR “flu” OR “LAV vaccine”) Filters: published in the last 10 years

(((recurrent OR frequent) AND respiratory AND infecti*)) AND (“Pneumococcal” OR “Influenza” OR “Vaccination*” OR “vaccin*” OR “Immunisation” OR “immunization” OR “Pneumovax” OR “Pnu-imune” OR “Prevenar” OR “PCV7” OR “7-valent” OR “seven valent” OR “PHiD-CV10” OR “PhiD-CV” OR “PCV-13” OR “13-valent” OR “thirteen valent” OR “flu” OR “LAV vaccine”) Filters: published in the last 10 years; Child: birth-18 years

(("Respiratory Tract Infections"[Mesh] AND (recurrent[TIAB] OR frequent[TIAB]))) AND (“Pneumococcal” OR “Influenza” OR “Vaccination*” OR “vaccin*” OR “Immunisation” OR “immunization” OR “Pneumovax” OR “Pnu-imune” OR “Prevenar” OR “PCV7” OR “7-valent” OR “seven valent” OR “PHiD-CV10” OR “PhiD-CV” OR “PCV-13” OR “13-valent” OR “thirteen valent” OR “flu” OR “LAV vaccine”) Filters: published in the last 10 years; Child: birth-18 years

**Nasal therapies with hyaluronic acid, thermal waters and resveratrol**

EMBASE

'respiratory tract infection'/exp AND (recurrent:ti,ab OR frequent:ti,ab) AND ([english]/lim OR [italian]/lim) AND ([infant]/lim OR [child]/lim OR [preschool]/lim OR [school]/lim OR [adolescent]/lim) AND [2009-2019]/py AND (‘saline irrigation’ OR ‘saline washing’ OR ‘hypertonic irrigation’ OR ‘hypertonic washing’ OR resveratrol OR glucan OR hyaluronic OR hyaluronate OR cucurbitacin)

(recurrent:ti,ab OR frequent:ti,ab) AND respiratory:ti,ab AND infecti*:ti,ab AND ([english]/lim OR [italian]/lim) AND [2009-2019]/py AND ([infant]/lim OR [child]/lim OR [preschool]/lim OR [school]/lim OR [adolescent]/lim) AND (‘saline irrigation’ OR ‘saline washing’ OR ‘hypertonic irrigation’ OR ‘hypertonic washing’ OR resveratrol OR glucan OR hyaluronic OR hyaluronate OR cucurbitacin)

(recurrent:ti,ab OR frequent:ti,ab) AND respiratory:ti,ab AND infecti*:ti,ab AND (child*:ti,ab OR pedia*:ti,ab OR paedia*:ti,ab) AND ([english]/lim OR [italian]/lim) AND [2009-2019]/py AND (‘saline irrigation’ OR ‘saline washing’ OR ‘hypertonic irrigation’ OR ‘hypertonic washing’ OR resveratrol OR glucan OR hyaluronic OR hyaluronate OR cucurbitacin)

PUBMED

((recurrent OR frequent) AND respiratory AND infecti* AND (child* OR pedia* OR paedia*)) OR (("Respiratory Tract Infections"[Mesh] AND (recurrent[TIAB] OR frequent[TIAB])) AND ("last 10 years"[PDat] AND (infant[MeSH] OR child[MeSH] OR adolescent[MeSH])))) AND (“saline irrigation” OR “saline washing” OR “hypertonic irrigation” OR “hypertonic washing” OR resveratrol OR glucan OR hyaluronic OR hyaluronate OR cucurbitacin)

**Modification of risk factors**

EMBASE

'respiratory tract infection'/exp AND (recurrent:ti,ab OR frequent:ti,ab) AND ([english]/lim OR [italian]/lim) AND ([infant]/lim OR [child]/lim OR [preschool]/lim OR [school]/lim OR [adolescent]/lim) AND ('virus infection'/exp OR 'virus infection' OR 'sibling'/exp OR sibling OR 'rural population'/exp OR 'rural population' OR 'air pollution'/exp OR 'air pollution' OR 'indoor air pollution'/exp OR 'indoor air pollution' OR 'passive smoking'/exp OR 'passive smoking' OR 'nursery school'/exp OR 'nursery school') AND [2009-2019]/py

(recurrent:ti,ab OR frequent:ti,ab) AND respiratory:ti,ab AND infecti*:ti,ab AND ([english]/lim OR [italian]/lim) AND ([infant]/lim OR [child]/lim OR [preschool]/lim OR [school]/lim OR [adolescent]/lim) AND ('virus infection'/exp OR 'virus infection' OR 'sibling'/exp OR sibling OR 'rural population'/exp OR 'rural population' OR 'air pollution'/exp OR 'air pollution' OR 'indoor air pollution'/exp OR 'indoor air pollution' OR 'passive smoking'/exp OR 'passive smoking' OR 'nursery school'/exp OR 'nursery school') AND [2009-2019]/py

(recurrent:ti,ab OR frequent:ti,ab) AND respiratory:ti,ab AND infecti*:ti,ab AND (child*:ti,ab OR pedia*:ti,ab OR paedia*:ti,ab) AND ([english]/lim OR [italian]/lim) AND ('virus infection'/exp OR 'virus infection' OR 'sibling'/exp OR sibling OR 'rural population'/exp OR 'rural population' OR 'air pollution'/exp OR 'air pollution' OR 'indoor air pollution'/exp OR 'indoor air pollution' OR 'passive smoking'/exp OR 'passive smoking' OR 'nursery school'/exp OR 'nursery school') AND [2009-2019]/py

PUBMED

(((recurrent OR frequent) AND respiratory AND infecti* AND (child* OR pedia* OR paedia*))) AND ((((((("Virus Diseases"[Mesh]) OR "Siblings"[Mesh]) OR "Rural Population"[Mesh]) OR "Air Pollution, Indoor"[Mesh]) OR "Air Pollution"[Mesh]) OR "Tobacco Smoke Pollution"[Mesh]) OR "Schools, Nursery"[Mesh])

(((recurrent OR frequent) AND respiratory AND infecti*)) AND ((((((("Virus Diseases"[Mesh]) OR "Siblings"[Mesh]) OR "Rural Population"[Mesh]) OR "Air Pollution, Indoor"[Mesh]) OR "Air Pollution"[Mesh]) OR "Tobacco Smoke Pollution"[Mesh]) OR "Schools, Nursery"[Mesh])

(("Respiratory Tract Infections"[Mesh] AND (recurrent[TIAB] OR frequent[TIAB]))) AND ((((((("Virus Diseases"[Mesh]) OR "Siblings"[Mesh]) OR "Rural Population"[Mesh]) OR "Air Pollution, Indoor"[Mesh]) OR "Air Pollution"[Mesh]) OR "Tobacco Smoke Pollution"[Mesh]) OR "Schools, Nursery"[Mesh])

**Antibiotic prophylaxis**

EMBASE:

'respiratory tract infection'/exp AND (recurrent:ti,ab OR frequent:ti,ab) AND ([english]/lim OR [italian]/lim) AND ([infant]/lim OR [child]/lim OR [preschool]/lim OR [school]/lim OR [adolescent]/lim) AND [2009-2019]/py AND ((antibiotic*:ti,ab OR bactericid*:ti,ab OR antibact*:ti,ab OR 'anti-bacterial':ti,ab) AND (prophyla*:ti,ab OR prevent*:ti,ab OR premedicat*:ti,ab) OR 'antibiotic prophylaxis'/exp OR ('antibiotic agent'/exp AND 'primary prevention'/exp))

(recurrent:ti,ab OR frequent:ti,ab) AND respiratory:ti,ab AND infecti*:ti,ab AND ([english]/lim OR [italian]/lim) AND [2009-2019]/py AND ([infant]/lim OR [child]/lim OR [preschool]/lim OR [school]/lim OR [adolescent]/lim) AND ((antibiotic*:ti,ab OR bactericid*:ti,ab OR antibact*:ti,ab OR 'anti-bacterial':ti,ab) AND (prophyla*:ti,ab OR prevent*:ti,ab OR premedicat*:ti,ab) OR 'antibiotic prophylaxis'/exp OR ('antibiotic agent'/exp AND 'primary prevention'/exp))

(recurrent:ti,ab OR frequent:ti,ab) AND respiratory:ti,ab AND infecti*:ti,ab AND (child*:ti,ab OR pedia*:ti,ab OR paedia*:ti,ab) AND ([english]/lim OR [italian]/lim) AND [2009-2019]/py AND ((antibiotic*:ti,ab OR bactericid*:ti,ab OR antibact*:ti,ab OR 'anti-bacterial':ti,ab) AND (prophyla*:ti,ab OR prevent*:ti,ab OR premedicat*:ti,ab) OR 'antibiotic prophylaxis'/exp OR ('antibiotic agent'/exp AND 'primary prevention'/exp))

PUBMED:

(recurrent OR frequent) AND respiratory AND infecti* AND (child* OR pedia* OR paedia*) AND (((antibiotic* OR bactericid* OR antibact* OR "anti-bacterial") AND (prophyla* OR prevent* OR premedicat*)) OR "Antibiotic Prophylaxis"[Mesh]) Filters: published in the last 10 years, Field: Title/Abstract

(recurrent OR frequent) AND respiratory AND infecti* AND (((antibiotic* OR bactericid* OR antibact* OR "anti-bacterial") AND (prophyla* OR prevent* OR premedicat*)) OR "Antibiotic Prophylaxis"[Mesh]) Filters: published in the last 10 years; Child: birth-18 years; Field: Title/Abstract

"Respiratory Tract Infections"[Mesh] AND (recurrent[TIAB] OR frequent[TIAB]) AND (((antibiotic* OR bactericid* OR antibact* OR "anti-bacterial") AND (prophyla* OR prevent* OR premedicat*)) OR "Antibiotic Prophylaxis"[Mesh]) Filters: published in the last 10 years; Child: birth-18 years

**Adeno/Tonsillectomy**

EMBASE

'respiratory tract infection'/exp AND (recurrent:ti,ab OR frequent:ti,ab) AND ([english]/lim OR [italian]/lim) AND ([infant]/lim OR [child]/lim OR [preschool]/lim OR [school]/lim OR [adolescent]/lim) AND ('adenoidectomy':ti,ab,kw OR 'tonsillectomy':ti,ab,kw OR 'surgery':ti,ab,kw) AND [2009-2019]/py

(recurrent:ti,ab OR frequent:ti,ab) AND respiratory:ti,ab AND infecti*:ti,ab AND ([english]/lim OR [italian]/lim) AND [2009-2019]/py AND ([infant]/lim OR [child]/lim OR [preschool]/lim OR [school]/lim OR [adolescent]/lim) AND ('adenoidectomy':ti,ab,kw OR 'tonsillectomy':ti,ab,kw OR 'surgery':ti,ab,kw)

(recurrent:ti,ab OR frequent:ti,ab) AND respiratory:ti,ab AND infecti*:ti,ab AND (child*:ti,ab OR pedia*:ti,ab OR paedia*:ti,ab) AND ([english]/lim OR [italian]/lim) AND [2009-2019]/py AND ('adenoidectomy':ti,ab,kw OR 'tonsillectomy':ti,ab,kw OR 'surgery':ti,ab,kw)

PUBMED

(((recurrent OR frequent) AND respiratory AND infecti* AND (child* OR pedia* OR paedia*))) AND ((("Adenoidectomy"[Mesh]) OR "Tonsillectomy"[Mesh]) OR "General Surgery"[Mesh])

(((recurrent OR frequent) AND respiratory AND infecti*)) AND ((("Adenoidectomy"[Mesh]) OR "Tonsillectomy"[Mesh]) OR "General Surgery"[Mesh])

(("Respiratory Tract Infections"[Mesh] AND (recurrent[TIAB] OR frequent[TIAB]))) AND ((("Adenoidectomy"[Mesh]) OR "Tonsillectomy"[Mesh]) OR "General Surgery"[Mesh]

**Appendix 2. Flowchart of included studies after literature review.**

Identification

**Records identified through database searching and after duplicates removed**

n= 4445

**Non-English languages**

n= 787

**Titles excluded**

n= 2760

Screening

**Abstract screened**

n= 898

**Abstract excluded**

n= 685

**Full-text articles assessed for eligibility**

n= 213

Eligibility

g

**Full-text articles excluded**

RRIs definition not reported

n=76

Full-text not available

n=41

Other topics

n=16

Included

**Full-text articles included in the analysis**

n= 80
